# A Stem-Cell-Derived Platform Enables Complete *Cryptosporidium* Development *In Vitro* and Genetic Tractability

**DOI:** 10.1016/j.chom.2019.05.007

**Published:** 2019-07-10

**Authors:** Georgia Wilke, Lisa J. Funkhouser-Jones, Yi Wang, Soumya Ravindran, Qiuling Wang, Wandy L. Beatty, Megan T. Baldridge, Kelli L. VanDussen, Bang Shen, Mark S. Kuhlenschmidt, Theresa B. Kuhlenschmidt, William H. Witola, Thaddeus S. Stappenbeck, L. David Sibley

**Affiliations:** 1 Department of Molecular Microbiology, Washington University School of Medicine, 660 S. Euclid Ave, St Louis, MO 63130, USA; 2 Department of Pathology and Immunology, Washington University School of Medicine, 660 S. Euclid Ave, St Louis, MO 63130, USA; 3 Department of Medicine, Division of Infectious Diseases, Edison Family Center for Genome Sciences and Systems Biology, Washington University School of Medicine, 660 S. Euclid Ave, St Louis, MO 63130, USA; 4 Department of Pathobiology, University of Illinois College of Veterinary Medicine, Urbana, IL 61802, USA

**Keywords:** stem cells, organoids, transcriptomics, pathway analysis, development, Mendelian genetics, meiosis, host-pathogen interactions

## Abstract

Despite being a frequent cause of severe diarrheal disease in infants and an opportunistic infection in immunocompromised patients, *Cryptosporidium* research has lagged due to a lack of facile experimental methods. Here, we describe a platform for complete life cycle development and long-term growth of *C. parvum in vitro* using “air-liquid interface” (ALI) cultures derived from intestinal epithelial stem cells. Transcriptomic profiling revealed that differentiating epithelial cells grown under ALI conditions undergo profound changes in metabolism and development that enable completion of the parasite life cycle *in vitro*. ALI cultures support parasite expansion > 100-fold and generate viable oocysts that are transmissible *in vitro* and to mice, causing infection and animal death. Transgenic parasite lines created using CRISPR/Cas9 were used to complete a genetic cross *in vitro*, demonstrating Mendelian segregation of chromosomes during meiosis. ALI culture provides an accessible model that will enable innovative studies into *Cryptosporidium* biology and host interactions.

## Highlights

• Air-liquid interface (ALI) cultivation of *Cryptosporidium* supports robust parasite growth
• Both asexual and sexual phases of the parasite complete development in ALI cultures
• ALI culture supports the production of *de novo* oocysts that can trigger an infection in mice
• *In vitro* crossing in ALI cultures opens up forward genetics for *Cryptosporidium*

## Graphical Abstract

**Figure:**
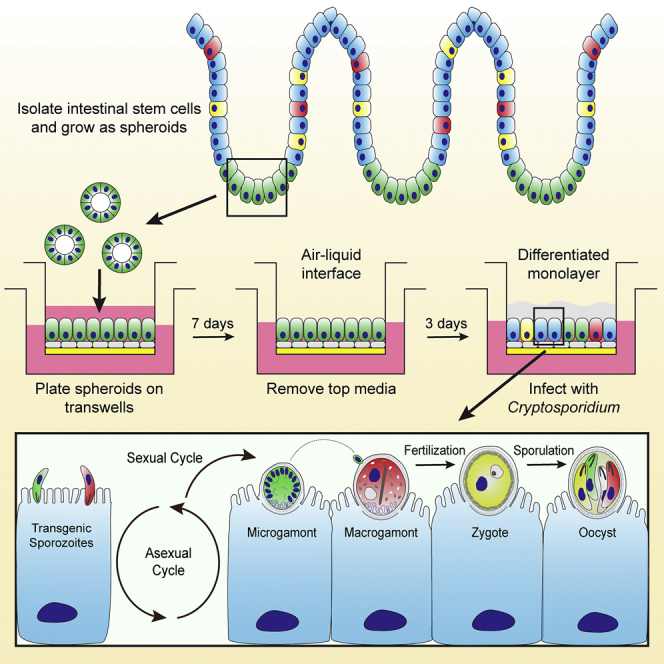

## Teaser

Wilke et al. describe an air-liquid interface (ALI) cultivation system that permits the efficient growth of *C. parvum in vitro*. ALI culture supports life cycle completion, thereby generating oocysts that can propagate *in vitro*, cause infection in mice, and enable *in vitro* genetic crosses, thus opening this system for future studies.

## Introduction

Cryptosporidium is a gastrointestinal parasite that causes long-term illness in immunocompromised patients (O'Connor et al., 2011) and contributes to malnourishment and impaired growth in children in low- to middle-income countries (Kotloff, 2017, Kotloff et al., 2013). Human infection is primarily caused by two species: *C. parvum* that is zoonotic and *C. hominis* that almost exclusively infects humans, while other species are much less frequently found in humans (Feng et al., 2018). Cryptosporidiosis is transmitted by an oral-fecal route, and the entire life cycle, consisting of asexual and sexual phases, takes place within intestinal enterocytes of a single host (Cevallos et al., 2000, Tzipori and Griffiths, 1998).

Biological investigations of *Cryptosporidium* have been hampered due to the lack of facile experimental platforms. Genetic modification of *C. parvum* using CRISPR/Cas9 requires propagation in immunocompromised mice (Vinayak et al., 2015), and stable transgenic parasites still cannot be selected or propagated *in vitro* due to the lack of robust cell culture platforms. Adenocarcinoma cell lines such as HCT-8 and Caco-2 support short-term growth, but the parasite does not complete its life cycle and replication ceases after a few rounds of asexual division (Upton et al., 1994). Because sexual recombination does not readily occur *in vitro*, little is known about the processes of sexual differentiation, fertilization, or meiosis in this parasite.

Studies using hollow fiber systems report complete life cycle development and passage of *C. parvum in vitro* (DeCicco RePass et al., 2017, Morada et al., 2016), but they require specialized equipment, are not easily scalable, and are not amenable to basic imaging techniques. A recent report that described the propagation of *C. parvum* in organoids derived from human intestinal epithelium demonstrates that complete development is possible in culture systems that mimic the parasite’s natural niche (Heo et al., 2018). However, this system requires microinjection of individual organoids with oocysts and hence is not readily scalable or directly amenable to experimental manipulation. An alternative method for creating long-term primary intestinal monolayers involves plating stem cells on transwells then removing the medium from the top chamber to form an “air-liquid interface” (ALI) (Wang et al., 2015). Here, we combined a modified ALI system with stem-cell derived spheroid cultures (Miyoshi et al., 2012, Miyoshi and Stappenbeck, 2013, VanDussen et al., 2015) to develop a system that supports long-term growth and complete development of *C. parvum* in vitro. We further demonstrate the utility of this system by generating transgenic parasites and performing an in vitro genetic cross, confirming the Mendelian nature of meiosis and opening forward genetics in *C. parvum*.

## Results

### ALI Monolayers Support Long-Term *C. Parvum* Infection *In Vitro*

**Figure 1:**
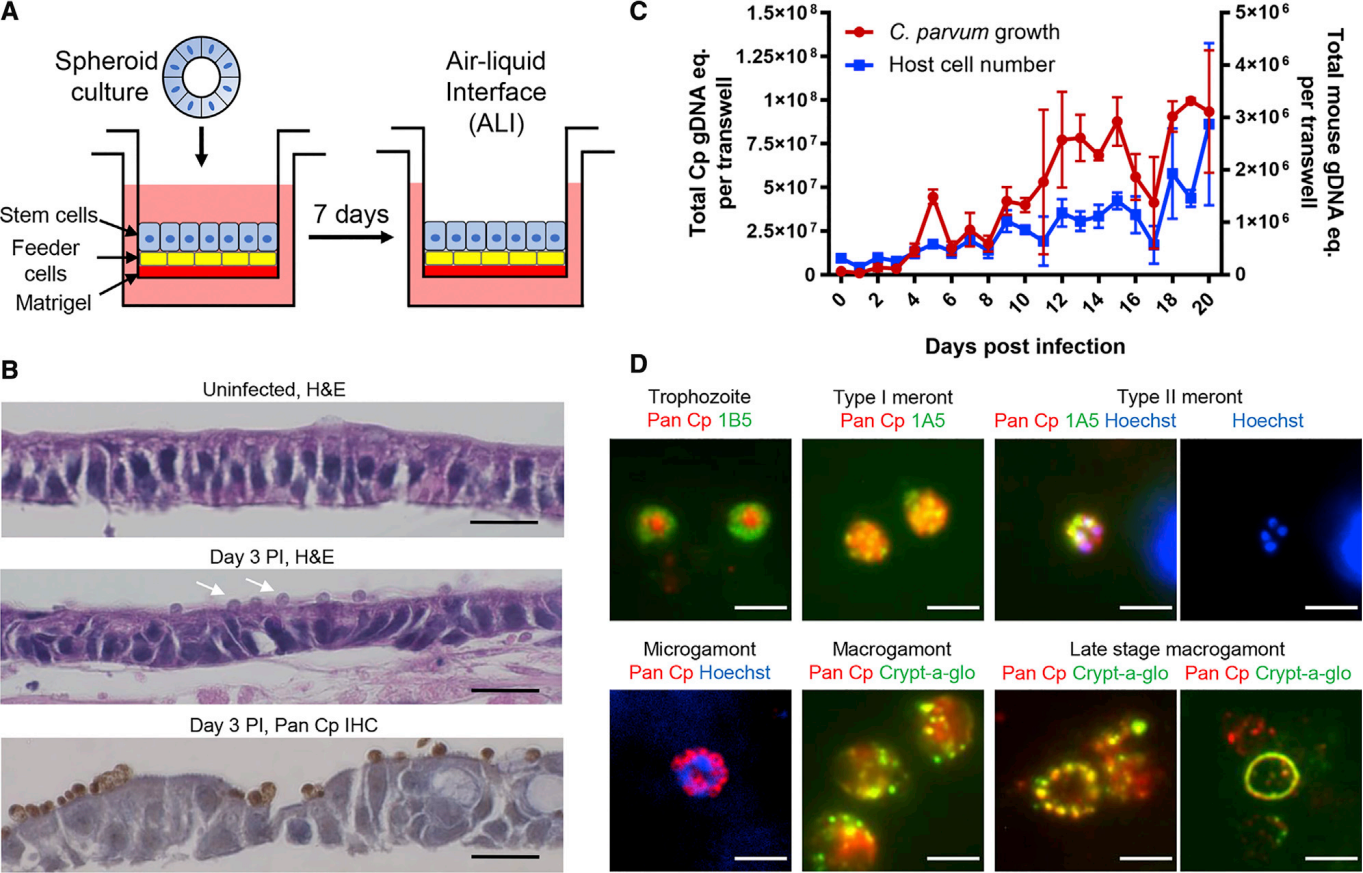
Development of an *In Vitro* System for Cultivation of *C. Parvum* (A) Model of air-liquid interface (ALI) culture method. See methods for details and Figures S1A and S1B for further description. (B) Histological examination of ALI cultures. Sections of ALI 3 days post-infection (PI) stained with hematoxylin and eosin (H&E), or rabbit pAb to detect *C. parvum* (referred to as Pan Cp) using immunohistochemistry (IHC). White arrows in middle panel highlight *C. parvum*. Scale bar, 20 μm. (C) Growth of *C. parvum* in ALI cultures infected 3 days post top medium removal with 2 × 10^5^ unfiltered *C. parvum* oocysts. The graph depicts qPCR measurement of *C. parvum* and mouse GAPDH equivalents (eq). Means ± S.D. of two transwells per time point from a representative experiment. See Figure S1C for the replicate experiment. (D) Detection of developmental stages of *C. parvum* in ALI cultures. Infected ALI transwells were fixed and stained with specified mouse mAbs (i.e., 1B5, 1A5) followed by goat antimouse IgG Alexa Fluor 488, Crypt-a-glo directly conjugated to FITC, or Pan Cp followed by goat anti-rabbit IgG Alexa Fluor 568. Hoechst staining for DNA. Scale bar, 3 μm. See also Figure S2.

We adopted previous methods for stem-cell derived cultures of mouse intestinal epithelial cells (mIECs) to differentiate monolayers on transwells under ALI conditions as a platform for *C parvum* growth *in vitro* (Figure 1A). Briefly, mIECs were amplified as spheroids (Miyoshi et al., 2012, Miyoshi and Stappenbeck, 2013, VanDussen et al., 2015), plated onto transwells that contained a matrigel coating and irradiated 3T3 cell feeder layer. mIECs monolayers were first grown submerged in conditioned medium (see STAR Methods) for 7 days, followed by removal of the top medium. Following the establishment of ALI conditions, cells in the monolayer underwent a burst in replication, as detected by staining with Ki-67 (Figure S1A). Additionally, cells began to express markers consistent with enterocyte and secretory cell lineages found in the small intestine, such as goblet cells and Paneth cells (Figure S1A). Electron microscopy of ALI monolayers processed at ∼ 1w-eek post top medium removal revealed the presence of a brush border and formation of tight junctions between epithelial cells at the apical side of the cells (Figure S1B). ALI cultures begin differentiating within a few days after top medium removal but do not reach full maturity until almost 14 days when the apical brush border became more prominent as detected with villin staining (Figure S1A).

To determine if ALI monolayers would support *C. parvum* growth, we infected monolayers with calf-derived oocysts and tracked parasite growth using microscopy and quantitative PCR (qPCR). Examination of H&E-stained sections of ALI monolayers showed a continuous monolayer of epithelial cells with a height comparable to that found *in vivo* (Figure 1B). Parasites were readily discernable at the apical surface of ALI monolayers examined by H&E staining or IHC (Figure 1B), consistent with their normal replicative niche in vivo, which has been described as intracellular but extracytoplasmic (Current and Reese, 1986). Parasite growth, as measured by qPCR, showed that *C. parvum* increased for at least 20 days post-infection, amplifying ∼100-fold from the initial infection (Figures 1C and S1C). This level of amplification is more than 10-fold higher than what we have observed in mIECs grown on conventional transwells without ALI conditions (Wilke et al., 2018) or that is reported in adenocarcinoma cell lines (Arrowood, 2002). Remarkably, the high parasite density in ALI cultures did not affect overall monolayer viability as evident from qPCR of mouse genomic DNA equivalents that increased over time (Figures 1C and S1C), and visual inspection of the infected transwells did not show signs of barrier breakdown or leakage.

### All Developmental Stages Of *C. Parvum* Occur in ALI Monolayers

To determine the extent of development of *C. parvum* in ALI cultures, we utilized a recently described panel of mAbs that stains intracellular stages of development (Wilke et al., 2018). Fluorescence microscopy of infected monolayers confirmed the appearance of asexual life cycle stages of *C. parvum*. Trophozoites were recognized by mAb 1B5, which labels a distinctive doughnut shape around trophozoites that may reflect the modified actin pedestal beneath the parasite (Wilke et al., 2018) (Figures 1D and S2A). Type I and II meronts were identified by mAb 1A5, which stains mature merozoites within meronts in a polarized manner (Wilke et al., 2018) (Figures 1D, S2B, and S2C). Sexual stages of *C. parvum* appeared by day 2 post infection and were abundant in the ALI cultures. Microgamonts were identified by their multiple small nuclei (up to 16) and separate cytoplasm detected by polyclonal antibody Pan Cp, which labels all forms of *C. parvum* (Wilke et al., 2018) (Figures 1D and S2D). Macrogamonts were identified by the presence of secretory vesicles detected with Crypt-a-glo (Figures 1D and S2E), a commercial FITC-conjugated mAb made against *C. parvum* oocyst outer wall proteins, or by OW50, a mAb that stains the oocyst wall (Arrowood and Sterling, 1989) (Figure S1D). These vesicular structures are known as wall-forming bodies (WFBs), and they contain proteins that are precursors of the oocyst wall (Spano et al., 1997). In late-stage macrogamonts, the WFBs coalesced into a ring at the perimeter of the parasite (Figures 1D, S1D, and S2G), which may represent a postfertilization stage before oocyst formation.

**Figure 2:**
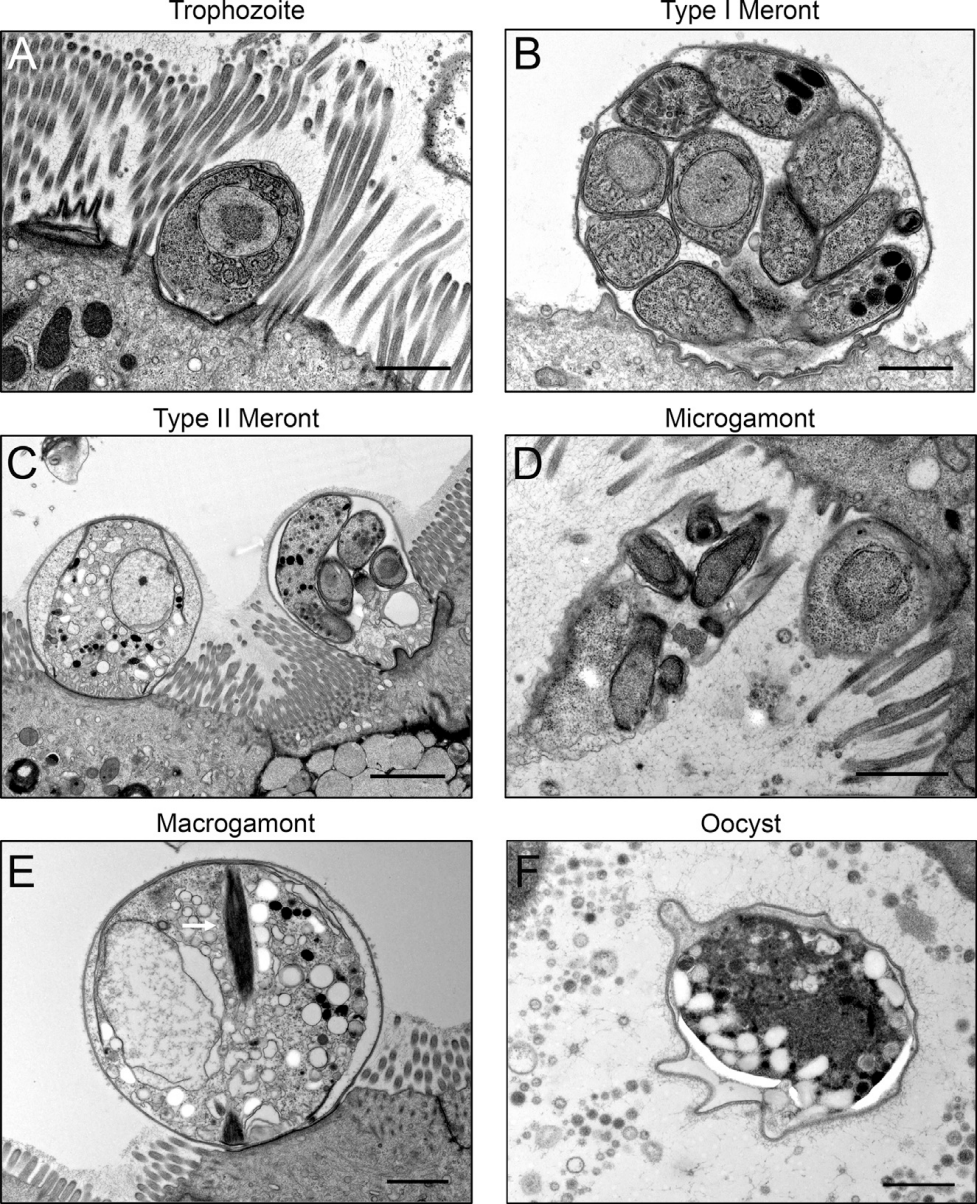
Electron Microscopy Images of *C. Parvum* Asexual Life Cycle Stages in ALI Cultures (A) Trophozoite adhering to enterocyte in ALI culture 3 dpi. (B) Type I meront on the apical surface of enterocyte in ALI culture 1 dpi. (C) Macrogamont (left) and type II meront (right, denoted with arrow) on the apical surface of enterocyte in ALI culture 4 dpi. (D) Microgamont is protruding from the surface of an enterocyte in ALI culture 3 dpi. Multiple bullet-shaped microgametes. (E) Macrogamont on the apical surface of enterocyte in ALI culture 4 dpi. Arrow highlights the presence of striated fiber. (F) Unsporulated oocyst shed from the surface of ALI culture 3 dpi. ALI transwell cultures were infected with 1 μm-filtered sporozoites 3 days post-medium removal. Scale bars for (A–B, D–F), 1 μm. Scale bar for (C) = 2 μm.

When examined by electron microscopy, most parasites were found in immature enterocytes (Figure 2), although occasionally they were seen in secretory cells. Trophozoites were recognized by their distinctive, single-nucleated morphology (Figure 2A), while type I meronts were contained in round parasitophorous vacuoles containing eight merozoites (Figure 2B). Type II meronts had taller, narrower, elongated vacuoles with four merozoites (Figure 2C), and microgamonts contained clusters of small bullet-shaped parasites (Figure 2D). Macrogamonts were identified based on their numerous lipid and glycogen vacuoles within the parasite cytoplasm, as well as the striated fiber previously described in macrogamonts (Wilke et al., 2018) (Figure 2E). Oocysts surrounded by a convoluted wall were also recognized in ALI cultures by electron microscopy, although a majority of these were unsporulated (Figure 2F).

### Robust *C. Parvum* Growth Requires ALI And Early Cell Differentiation

**Figure 3:**
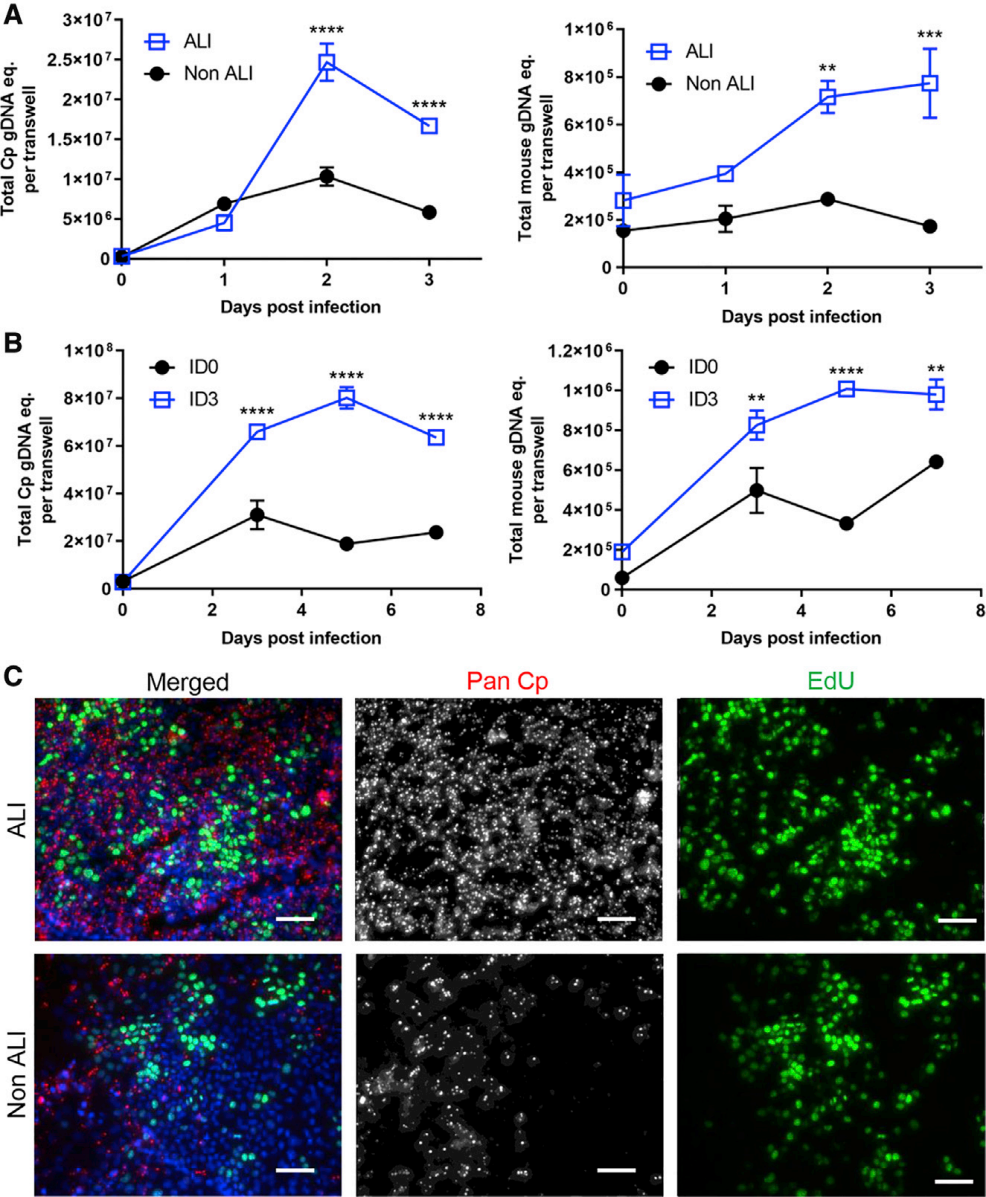
Effects of Timing and Differentiation on *C. Parvum* Growth in ALI Cultures (A and B) Comparison of *C. parvum* growth. (A) Transwell cultures infected three days post top medium removal (ALI) versus continuous top medium (non-ALI). (B) ALI cultures infected with 2 × 10^5^ unfiltered oocysts at day 0 (ID0) versus day 3 (ID3) post medium removal. Left, *C. parvum* genomic DNA equivalents (Cp gDNA eq.). Right, mouse genomic DNA equivalents (mouse gDNA eq.) measured by qPCR. Means ± S.D. from two transwells per time point from a representative experiment. Two-way ANOVA comparing the means of each group at each time point, corrected for multiple comparisons using the Sidak method, ^∗∗^p < 0.01, ^∗∗∗^p < 0.001, ^∗∗∗∗^p < 0.0001. See also Figure S3 for replicate experiments. (C) Microscopic examination of host cell and *C. parvum* proliferation in ALI versus non-ALI transwell cultures 3 dpi after an 8-h incubation with 10 μM EdU in the bottom chamber medium. *C. parvum* was stained with Pan Cp followed by goat anti-rabbit IgG Alexa Fluor 568, DNA stained with Hoechst. Scale bars, 50 μm.

To determine whether ALI conditions were required for *C. parvum* growth, we compared *C. parvum* proliferation in infected “submerged” mIEC monolayers grown on transwells without removal of the top medium (non-ALI) to ALI transwells infected 3 days after removal of the top medium. *C. parvum* grew significantly better in ALI transwells compared to non-ALI transwells (Figures 3A and S3A), while host cell viability during infection was improved in ALI transwells compared to non-ALI transwells despite the higher infection levels in the former (Figures 3A and S3A).

To determine the influence of cell differentiation on *C. parvum* growth, we infected transwells on day 0 of ALI initiation vs. day 3 postmedium removal, when monolayers first begin to show signs of differentiation (Figure S1A). *C. parvum* grew significantly better in transwells infected 3 days after ALI initiation compared to day 0 ALI cultures, which only supported low levels of expansion (Figures 3B and S3B). Similar to the ALI vs. non-ALI experiment, host cell viability during infection was improved in the transwells infected on day 3 (Figures 3B and S3B).

The above findings suggested that enhanced *C. parvum* growth was tied to improved host cell viability and proliferation triggered by ALI growth conditions. To visualize cell proliferation during infection, we pulsed parallel sets of infected, submerged non-ALI and 3-day ALI cultures with EdU in the bottom chamber medium for 8 h. Microscopy showed higher numbers of both *C. parvum* and proliferating cells (EdU positive) in transwells infected 3 days after ALI initiation compared to the submerged non-ALI transwells (Figure 3C). Although *C. parvum* was primarily found in non-EdU positive cells, this pattern was not exclusive (Figure 3C). In separate experiments, we observed that ALI monolayers are permissive to infection from days 3–6, but that infection is less readily established after this time period, perhaps due to the dramatic increase in mucus production that is observed at later times points (Figure S1A). As such, for all subsequent experiment we focused on the optimal conditions of infecting ALI cultures 3 days after removal of the top medium.

### ALI Culture Leads to Cellular Replication, Altered Metabolism, and Differentiation

**Figure 4:**
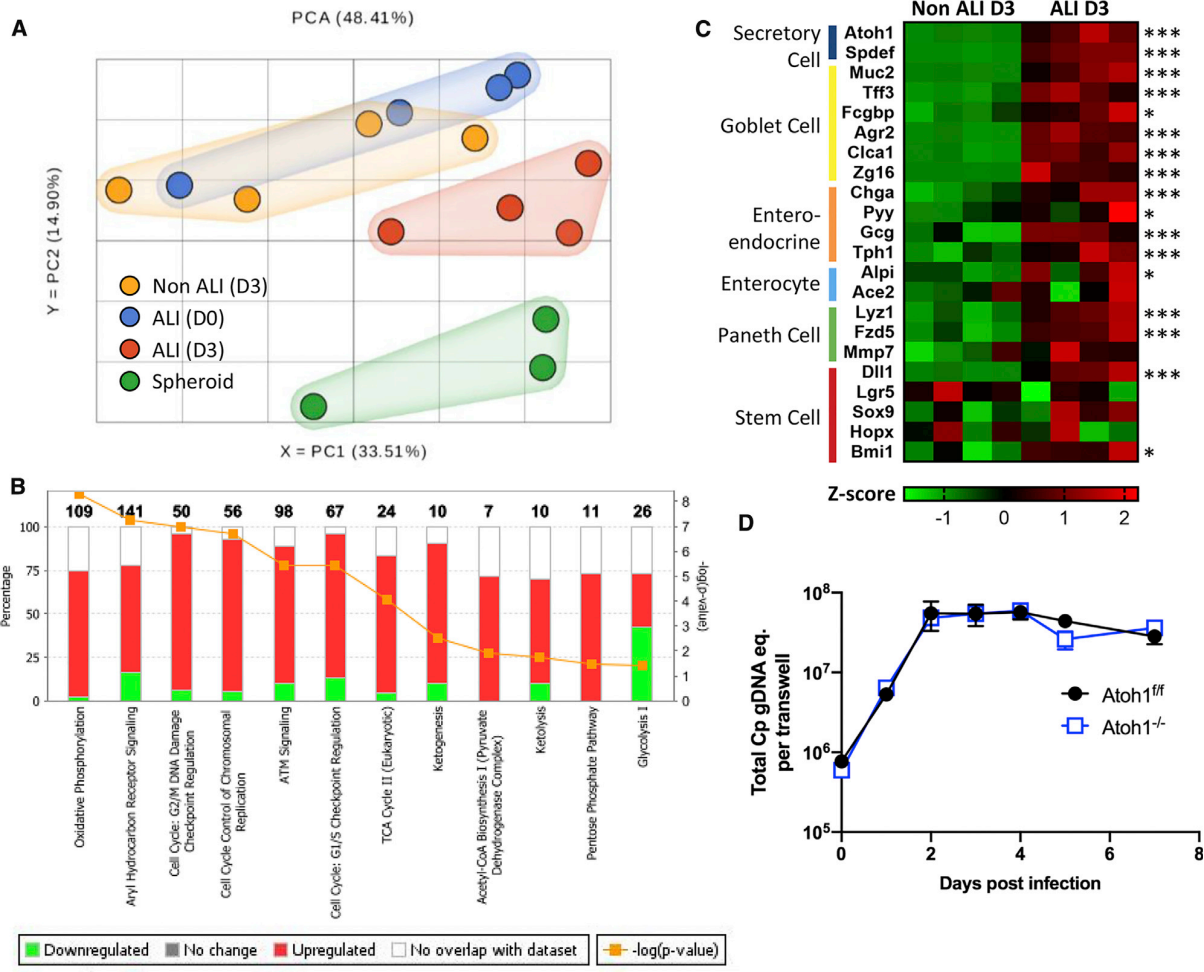
Pathway Analysis of Transcriptomes from Stem Cells and Differentiating ALI Cultures (A) Principal-component analysis (PCA) of normalized expression values for all genes in stem cell spheroids (green), transwells on day 0 (ALI D0, blue) or day 3, either after medium removal (ALI D3, red) or with continuous medium (non-ALI D3, yellow). Two independent experiments with two replicates per experiment except for the spheroid group, in which one technical replicate was removed from downstream analyses due to poor quality reads (see STAR Methods). (B) Bar chart of the percentage of genes upregulated (red) or downregulated (green) in day 3 ALI versus non-ALI samples for the most significant pathways (p < 0.05) under the “generation of precursor metabolites and energy” and “cell cycle regulation” pathways, respectively. Numbers in bold indicate the total number of genes per pathway. Performed in ingenuity pathway analysis (IPA) using expression values for all genes as input. See also Figure S4. (C) Heatmap of expression differences between day 3 non-ALI and ALI samples. The Z-score indicates the number of standard deviations that the expression value of each sample is above (red) or below (green) the mean expression value for all samples in the analysis. FDR-corrected p values for differential expression were determined in Partek using gene specific analysis (GSA): ^∗^q < 0.05, ^∗∗∗^q < 0.001. (D) Comparison of *C. parvum* growth in *Atoh1*^*−/−*^ knockout (blue) or matched floxed Atoh1^f/f^ control (black) ALI cultures infected with 2 × 10^5^ unfiltered oocysts. *C. parvum* genomic equivalents (eq.) measured by qPCR for the *C. parvum* GAPDH gene. Means ± S.D. from two transwells per time point from a representative experiment. See also Figure S5.

To determine the underlying changes that occur in ALI cultures following removal of top medium, we compared RNA-seq transcriptomes of mouse ileal cells replicating as spheroid cultures to day 0 ALI (before top medium removal), day 3 non-ALI (submerged transwell cultures), and day 3 ALI cultures. Analysis of gene expression differences by principle component analysis indicated that spheroid cultures were highly different from the rest of the samples (Figure 4A), as might be expected from the differences in growth conditions (i.e., they are embedded in Matrigel in a 3-D system instead of grown on transwells). In comparing cultures grown on transwells, day 3 ALI samples clustered in a distinct group, while non-ALI day 3 and ALI day 0 samples were largely overlapping (Figure 4A). To more closely examine these changes, we compared the expression of all genes between day 3 non-ALI and ALI samples using Ingenuity Pathway Analysis (IPA) to define pathways that were significantly different (p < 0.05) between growth conditions based on concordant changes among genes within similar pathways. A subset of the most prominently differentially expressed metabolism and cell cycle pathways is illustrated in Figure 4B, highlighting the changes in glycolysis (down regulated in day 3 ALI) vs. oxidative phosphorylation and the TCA cycle (up regulated in day 3 ALI) and cell cycle and replication (up regulated in day 3 ALI). Additional changes in gene expression profiles are highlighted in Figure S4.

Histological studies indicated that secretory cell lineages started to appear as early as day 3 in ALI cultures (Figure S1A); therefore, we analyzed a subset of genes related to epithelial cell differentiation including markers for secretory cells (i.e., Paneth, goblet, and enteroendocrine cells). Genes related to the development of these intestinal cell lineages were significantly upregulated in day 3 ALI versus day 3 non-ALI cultures (Figure 4C). Additionally, stem cell markers Lgr5 and HopX were slightly decreased in day 3 ALI, while Bmi1 was elevated (Figure 4C), as were markers Sox9 and Dll1, which are highly expressed in transient amplifying cells that emerge from the crypt after being produced from intestinal stem cells (Bankaitis et al., 2018). These findings are consistent with the observation that ALI cultures undergo differentiation, yet still contain proliferative cells that are labeled by EdU and Ki-67 (Figures 3C and S1A). Finally, because the transcription factor ATOH1 is significantly upregulated in day 3 ALI (Figure 4C), we tested the reliance of *C. parvum* growth on secretory cells that are dependent on this transcription factor for development (i.e. Paneth, goblet, enteroendocrine cells) (Shroyer et al., 2007). Because Atoh1 knockout mice die shortly after birth (Yang et al., 2001), we generated a spheroid line from a floxed Atoh1 mouse then excised the Atoh1 gene by the introduction of Cre-recombinase in vitro (Figure S5). Inoculation of wild type (Atoh1^f/f^) and Atoh1 null (*Atoh1*^*−/−*^) ALI transwells revealed that *C. parvum* grew robustly in both cell lines (Figure 4D), indicating that parasite growth was not dependent on the secretory cell lineages.

### ALI Monolayers Support Oocyst Development

**Figure 5:**
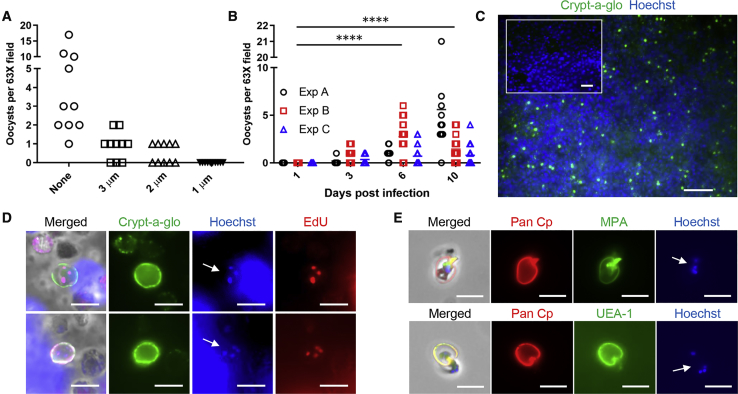
*In vitro* production of oocysts in ALI culture (A) Effect of filtration on removal of residual *C. parvum* oocysts. Oocysts were excysted, filtered using the indicated pore sizes, added to PLL-coated coverslips, and stained with Crypt-a-glo directly conjugated to FITC. The number of residual oocysts found in filtered samples were counted from replicate 63× fields from a representative experiment. (B) Detection of *C. parvum* oocysts in ALI cultures. Transwells were infected on day 3 post top medium removal with 1 μm-filtered sporozoites. On specified days post infection, transwells were fixed and stained with Crypt-a-glo directly conjugated to FITC and Pan Cp, detected with goat anti-rabbit IgG Alexa Fluor 568. Oocyst numbers per 63× field were counted for three independent experiments (Exp A, Exp B, Exp C). The combined mean of each time point was compared to the combined mean of day 1 using a two-way ANOVA corrected for multiple comparisons using the Dunnett method, ^∗∗∗∗^ p < 0.0001. (C) Low magnification (20×) image of oocyst development in ALI transwells infected with 1 μm-filtered sporozoites 10 days or 1 day post infection (inset). Transwells were stained with Crypt-a-glo directly conjugated to FITC to detect oocysts. DNA stained with Hoechst. Scale bars, 50 μm. (D) Labeling of newly formed oocysts with EdU. ALI transwells were infected on day 3 post top medium removal with 1 μm-filtered sporozoites. On day 2 post infection, 10 μM EdU was added to the bottom chamber medium and incubated overnight. Transwells were fixed and EdU was labeled using the Click-iT EdU Alexa Fluor 488. *C. parvum* was labeled with Crypt-a-glo directly conjugated to FITC, DNA stained with Hoechst (white arrows denote nuclei). Scale bars, 5 μm. (E) Lectin labeling of oocysts. Transwells were infected with 1 μm-filtered sporozoites and bleached on day 3 post infection, adhered to PLL-coated coverslips, and stained with Pan Cp followed by goat anti-rabbit Alexa Fluor 568, Hoechst, and either lectins MPA or UEA-1 directly conjugated to FITC. DNA stained with Hoechst (white arrows denote nuclei). Scale bars = 5 μm.

To determine if oocysts were being produced de novo in the ALI monolayers, we infected transwells with excysted sporozoites passed through filters with a 1 μm pore size, a step we determined was essential to remove all residual input oocysts (Figure 5A). To visualize oocyst development, we stained the monolayers with Crypt-a-glo and counted the number of oocysts per field. Oocysts began to appear by day 3 and varied in frequency within different regions of the culture (i.e., 1–10 oocysts per high powered field) and across different experiments (Figure 5B), although occasionally they were quite numerous (Figure 5C). Based on the frequencies reported in Figure 5B, we estimate that each transwell contained between 100-1,000 oocysts starting 3 days post infection. However, oocyst numbers went through periodic cycles over time (Figure 5B), suggesting there are successive rounds of production followed by hatching and reinitiation of infection. To confirm the oocysts observed by staining were indeed newly produced in culture, we added EdU to the culture medium overnight and examined incorporation into the nuclei of sporozoites. We readily identified oocysts containing four EdU-positive sporozoite nuclei in the ALI monolayers (Figure 5D), confirming that the oocysts observed in ALI cultures replicated and sporulated in vitro and were not the result of contamination from the original inoculum.

It has been shown previously that *C. parvum* oocysts bind the GalNAc-binding lectin *Maclura pomifera* agglutinin (MPA) and the fucose-binding lectin *Ulex europaeus* agglutinin I (UEA-1) (Chatterjee et al., 2010). To confirm that the in vitro-produced oocysts shared these characteristics, we isolated the oocysts from the ALI monolayers by bleaching the monolayers to remove cell debris and intracellular parasites, then plated the remaining material on poly-L-lysine-coated coverslips. We stained the material with MPA and UEA-1 lectins and observed lectin-positive oocysts (Figure 5E), confirming the oocysts produced in vitro shared the normal characteristics of mature oocysts.

### Oocysts Produced in Vitro are Infectious to Mice

**Figure 6:**
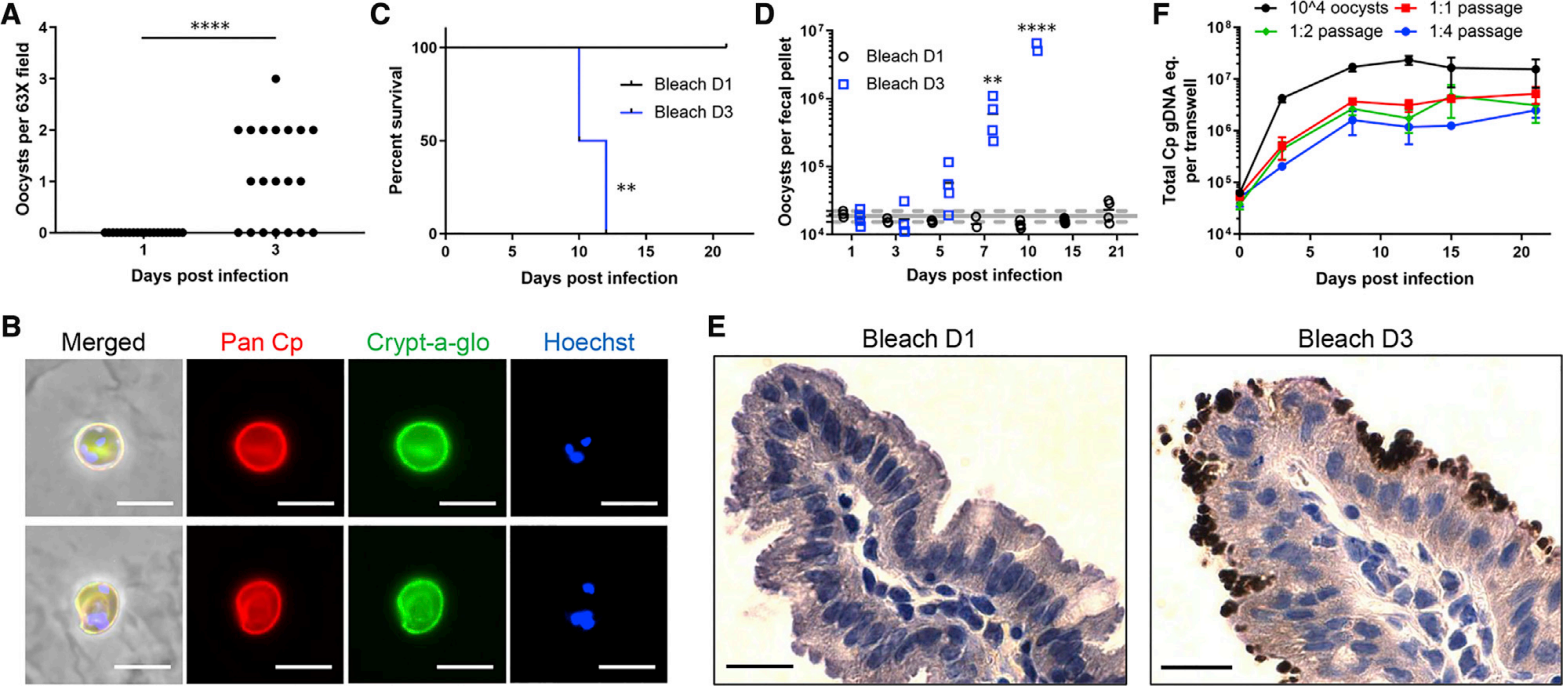
Infectivity of oocysts produced in ALI cultures (A) Detection of oocysts in ALI cultures treated with bleach. ALI transwell cultures were infected with 1 μm-filtered sporozoites. On days 1 and 3 postinfection, transwells were bleached, washed, pelleted, adhered to PLL-coated coverslips, and stained with Pan Cp followed by goat anti-rabbit Alexa Fluor 568 and Crypt-a-glo directly conjugated to FITC. Each data point is the number of oocysts in a single 63X field from a single representative experiment. Data were analyzed using a Mann-Whitney U test. ^∗∗∗∗^ p < 0.0001. See also Figure S6 for replicate experiment. (B) Images of bleached oocysts isolated from ALI cultures at day 3 postinfection as described in (A). Oocysts were stained with Pan Cp followed by goat anti-rabbit IgG Alexa Fluor 568, Crypt-a-glo directly conjugated to FITC, and Hoechst. Scale bars, 5 μm. (C and D) Infectivity of bleached ALI monolayers for mice. ALI transwell cultures were infected with 1 μm-filtered sporozoites. On days 1 and 3 postinfection, transwells were bleached, washed, and the material was orally gavaged into naïve *Ifngr1*^*−/−*^ mice. (C) Survival curves of the mice analyzed using the log-rank (Mantel-Cox) test, ^∗∗^p < 0.01. (D) Number of oocysts per fecal pellet as measured by qPCR for the *C. parvum* GAPDH gene. The gray line represents the mean value of both groups for day 1; the dotted lines are the day 1 standard deviations. Data was analyzed using a two-way ANOVA comparing the means of Bleach D1 vs Bleach D3 across all time points, corrected for multiple comparisons using the Sidak method, ^∗∗^ p < 0.01, ^∗∗∗∗^ p < 0.0001. See also Figures S6B and S6C for replicate example. (E) Histological examination of mice infected with bleached material from ALI cultures. Sections of small intestine stained with Pan Cp and revealed by IHC. Day 1 recipient was sacrificed on day 30 postgavage (image on left), day 3 recipient was sacrificed on day 9 postgavage (image on right). Scale bars, 20 μm. (F) Passage of bleached ALI monolayers. ALI transwell cultures were infected with 1 μm-filtered sporozoites. Bleached day 3 ALI cultures were used to infect naïve ALI monolayers at a 1:1, 1:2 or 1:4 passage ratio. In parallel, ALI cultures were infected with 10^4^ calf-derived oocysts. Outgrowth of passaged *C. parvum* was monitored by qPCR. Data plotted as mean ± S.D. from two transwells per time point. See Figure S6D for replicate experiment.

To determine whether the oocysts produced in vitro were viable and infectious, we infected ALI monolayers with filtered sporozoites and then treated the monolayers with bleach to kill all parasites except oocysts (Fayer, 1995). Bleached material stained with Crypt-a-glo showed that day 3 ALI cultures contained numerous oocysts, while day 1 cultures were negative (Figures 6A, 6B, and S6A). We then infected *Ifngr1*^*−/−*^ mice, which are highly susceptible to *C. parvum* (Griffiths et al., 1998, von Oettingen et al., 2008) with bleached material from day 1 and day 3 cultures by oral gavage. Mice that received day 1 material survived for the duration of the experiment (Figures 6C and S6B) and did not show any evidence of oocyst shedding above the background signal in the assay (Figures 6D and S6C). Conversely, mice that received day 3 material began shedding oocysts by day 5 postgavage, and the number of oocysts increased almost two logs before the animals died (Figures 6D and S6C), which occurred by day 12 postgavage (Figures 6C and S6B). Consistent with the shedding results, intestinal tissue sections from mice that received the day 3 material showed robust infection by anti-*C. parvum* IHC, but no sign of infection was seen in mice inoculated with day 1 material (Figure 6E). We also initiated infection of new ALI monolayers with bleached oocysts obtained from day 3 ALI cultures, including dilutions of the material, which readily expanded at a similar rate to cultures initiated with calf-derived oocysts (Figures 6F and S6D).

### Generation of Stable Transgenic Parasites

**Figure 7:**
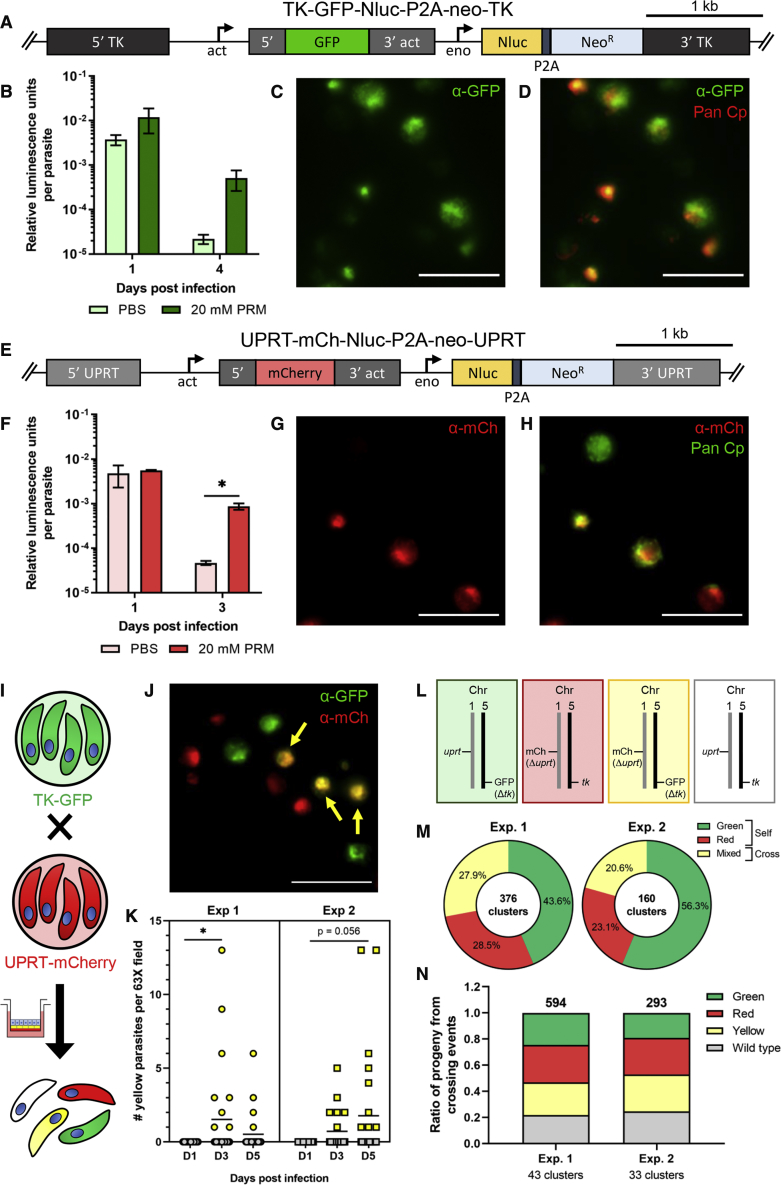
ALI Transwells support genetic crosses of *C. parvum* in vitro (A) Diagram of targeting construct designed to replace the endogenous *tk* locus (cgd5_4440) with GFP and Nluc-P2A-Neo^R^ cassette. (B) Relative luminescence normalized to total number of parasites in ALI transwells at 1 and 4 days post infection (dpi). Transwells were infected with ∼1 × 10^7^ unfiltered sporozoites that were electroporated with the TK-GFP-Nluc-P2A-neo-TK reporter and a Cas9 plasmid with a TK gRNA. Transwells were cultured in medium containing PBS (light green) as a control or 20 mM paromomycin (PRM, dark green). Data plotted as mean ± S.D. from two transwells per time point from a representative experiment. Nonsignificant (p = 0.11), unpaired Student’s t test between PBS and PRM-treated transwells 4 dpi. (C) Image of whole-mount ALI transwells 5 dpi with transfected *C. parvum* from same experiment as (B) stained with anti-GFP followed by goat anti-rabbit IgG Alexa Fluor 488. Scale bar, 10 μm. (D) Merged image of (C) with a Pan Cp polyclonal antibody, which recognizes all *C. parvum* stages, followed by goat antirat IgG Alexa Fluor 568. Scale bar, 10 μm. (E) Diagram of targeting construct designed to replace the endogenous *uprt* locus (cgd1_1900) with mCherry and Nluc-P2A-Neo^R^ cassette as (A). (F) Relative luminescence normalized to total number of parasites in ALI transwells at 1 and 3 dpi. Transwells were infected with ∼1 × 10^7^ unfiltered sporozoites per transwell that were electroporated with the UPRT-mCh-Nluc-P2A-neo-UPRT reporter and a Cas9 plasmid with a UPRT gRNA. Transwells were cultured in medium containing PBS (pink) as a control or 20 mM PRM (red). Data plotted as mean ± S.D. from two transwells per time point from a representative experiment. ^∗^p < 0.05, unpaired Student’s t test between PBS and PRM-treated transwells 3 dpi. (G) Image of whole-mount ALI transwells 5 dpi with transfected *C. parvum* from same experiment as (G) stained with anti-mCherry followed by goat antirat Alexa Fluor IgG 568. Scale bar, 10 μm. (H) Merged image of (G) with Pan Cp polyclonal antibody followed by goat anti-rabbit IgG Alexa Fluor 488. Scale bar, 10 μm. (I) Diagram of crossing experiments in vitro in which TK-GFP oocysts (green) and UPRT-mCh oocysts (red) are added to the same ALI Transwell. (J) ALI transwells were infected with TK-GFP and UPRT-mCh oocysts (2.5 × 10^4^ unfiltered oocysts per strain) and stained 5 dpi with anti-GFP followed by goat anti-rabbit Alexa Fluor IgG 488 and anti-mCherry followed by goat antirat Alexa Fluor IgG 568. Parasites that express both markers (yellow arrows). Scale bar, 10 μm. (K) ALI transwells were infected with TK-GFP and UPRT-mCh oocysts (5 × 10^4^ unfiltered oocysts per strain) and stained with antibodies as in (J). Images of 13 – 26 fields at 63× were acquired for 1–2 transwells per time point, and the number of parasites expressing both markers per field was counted for two independent experiments. ^∗^p < 0.05, nonparametric Kruskal-Wallis test with a Dunn’s multiple comparison test. (L) Predicted segregation patterns for chromosomes following meiosis. (M) ALI transwells were infected as in (K). After one round of oocyst formation (3 dpi), transwells were scraped, bleached, and added to HCT-8 monolayers for 15 – 18 hr post infection (hpi), then fixed and stained with the same antibodies as (J) plus mouse monoclonal 1E12 followed by goat antimouse IgG Alexa Fluor 647 to detect wild type parasites. Pie chart shows the percentage of all green, all red, or mixed clusters for two independent experiments. Observed ratios significantly deviate from the expected 50% for mixed clusters, 25% each for green and red (p < 0.001, Chi-square test). (N) Bar graph of the ratio of parasites with each phenotype to the total number of parasites within the “mixed” clusters from the same two independent experiments as in (M). Observed ratios do not significantly deviate from the expected 25% for each phenotype (p = 0.14 for Exp. 1 and p = 0.09 for Exp. 2, Chi-square test). See also Figure S7.

To generate transgenic parasite strains, we modified a previously described nanoluciferase (Nluc) reporter fused to neomycin resistance (NeoR) (Vinayak et al., 2015) by inserting a P2A skip peptide to increase luciferase expression and by adding GFP driven by the *C. parvum* actin promoter (Figures 7A and S7C). Excysted sporozoites were electroporated with the TK-GFP-Nluc-P2A-neo-TK plasmid and a newly constructed Cas9 plasmid (Table S2) containing a *tk* guide RNA sequence (Vinayak et al., 2015), then added to ALI transwells. Eight h post infection (p.i.), extracellular parasites were washed from the top of the transwells, and bottom medium was replaced with medium containing 20 mM paromomycin (PRM) for selection or PBS control. On the first day p.i., both drug-treated and nontreated transwells had high luminescence values, indicative of transient expression (Figure 7B). By the fourth day of selection, luminescence values per parasite were 23-fold higher on average in PRM vs. PBS-treated transwells (Figure 7B), although this difference was not statistically significant. Staining with a Pan Cp polyclonal antibody that recognizes all stages of *C. parvum* showed that most parasites in the culture expressed GFP, indicating that selection enriched substantially for transgenic parasites (Figure 7D).

The *C. parvum* genome contains two uracil phosphoribosyltransferase (UPRT) enzymes capable of converting scavenged host uridine into uridine monophosphate (Striepen et al., 2004). For a second fluorescent parasite strain, we designed a targeting construct to replace one of the *C. parvum* UPRT genes (cgd1_1900) with a mCherry reporter upstream of the Nluc-P2A-neo cassette described above (Figures 7E and S7F). Sporozoites electroporated with the UPRT-mCh-Nluc-P2A-neo-UPRT plasmid and a Cas9 plasmid with a UPRT gRNA were selected for growth in ALI transwells treated with 20 mM PRM. We detected an 18-fold increase in relative luminescence per parasite on average in PRM vs. PBS-treated transwells by 3 dpi, a difference that was statistically significant (Figure 7F). Immunofluorescence staining confirmed that most of the parasites expressed the mCherry transgene (Figure 7H). To generate larger numbers of oocysts, we passaged the mixed transgenic populations in immunocompromised mice under drug selection (16 g/L PRM in the drinking water) and then purified the oocysts from infected fecal pellets (Figures S7B–S7G). Using primers located inside and outside the deleted genes, we confirmed that both the TK-GFP and UPRT-mCherry strains had the correct insertion into the genomic loci and were lacking the *tk* and *uprt* genes, respectively (Figures S7E and S7H), indicating that the populations are pure transgenic lines.

### Crossing Reporter Strains *In Vitro* Produces Viable, Recombinant Oocysts

Since the GFP and mCherry reporters were inserted into different chromosomes (5 and 1, respectively), cross-fertilization in vitro should result in recombinant progeny expressing both reporters following the first round of oocyst formation (Figure 7I). We infected ALI transwells with equal numbers of TK-GFP and UPRT-mCherry parasites, then examined cultures by microscopy at 3 to 5 dpi to detect the presence of parasites expressing both reporters (Figure 7J). In two independent experiments, yellow parasites were absent at 1 dpi but appeared by 3 or 5 dpi, consistent with the time needed for completion of the life cycle (Figure 7K).

Following crossing between TK-GFP and UPRT-mCherry parasites, oocysts should contain sporozoites of different genotypes, based on independent segregation of the chromosomes (Figure 7L). To test this prediction, we infected ALI transwells with equal numbers of TK-GFP and UPRT-mCherry sporozoites, then enriched for oocysts at 3 dpi by bleaching transwells. We recovered and plated the oocysts on a human adenocarcinoma cell line (HCT-8) at a low MOI such that parasites descended from a single oocyst would form separate clusters as they grew out. If the oocyst was a result of self-fertilization, then all parasites in a cluster were expected to express the same color (either green or red), whereas cross-fertilization events should produce clusters of parasites with mixed phenotypes. After a single round of merogony during the outgrowth phase (15–18 hpi), we observed that 20.6%–27.9% of clusters had parasites with mixed phenotypes, depending on the experiment (Figure 7M), confirming that they arose from the product of outcrossing. This value is significantly lower than expected, presumably due to the localized nature of infection in the transwells that limited outcrossing rather than an intrinsic defect in fertilization, as both lines productively underwent self-fertalization in mice (Figure S7). The contents of oocysts formed from out-crossing are expected to initially express both markers and to be yellow as the sporozoites are formed from a commonly inherited cytoplasm (Current and Reese, 1986). However, as parasites grow out from individual sporozoites, their phenotype (i.e., colors) will reflect the genotypes they inherited in the cross, as diagrammed in Figure 7L. Importantly, the distribution of different genotypes, and hence color patterns, among parasites from these mixed clusters did not significantly deviate from the expected frequency of 25% for each phenotype (Figure 7N).

## Discussion

Our findings establish that cultivation of stem-cell derived intestinal epithelial cells under ALI conditions enables complete development of the *C. parvum* life cycle and provides a platform to investigate host-parasite interactions using forward and reverse genetics. In a separate report (Wang and Stappenbeck, unpublished), the use of ALI culture conditions as employed here is shown to induce a critical population of HopX-positive stem cells that drives regeneration, differentiation, and restores gut homeostasis. Following the switch to ALI culture conditions, mIEC monolayers underwent dramatic expansion and differentiation from stem cells to resemble cell lineages normally found in the intestine. Expansion of parasites did not appear to adversely influence the integrity of the monolayer, perhaps because of the continual renewal of cells from stem cells. Although the infection was seen in all cell types, *C. parvum* grew equally well in *Atoh1*^*−/−*^ cultures, which lack the capacity to develop the secretory cell lineages (Bankaitis et al., 2018). Similarly, *C. parvum* infection is not restricted to a specific cell type in the intestine, although it occurs primarily in enterocytes (Moore et al., 1995, Pereira et al., 2002, Thulin et al., 1994).

Analysis of gene expression data revealed profound changes in metabolism in ALI cultures to favor oxidative phosphorylation over glycolysis. This change is intriguing as adenocarcinoma cells, which only support limited growth of *C. parvum*, are also highly glycolytic, a phenotype common to many cancer cells known as the Warburg effect (Potter et al., 2016). When mIECs were cultured in the presence of the top medium, this metabolic shift was not evident, and they only supported modest growth. The data suggest that changes in host cell metabolism are key for supporting optimal growth and complete development of *C. parvum*, although further studies would be needed to test this hypothesis. Collectively, ALI monolayers likely support *C. parvum* growth in vitro because they resemble the in vivo niche of the parasite in terms of gene expression, metabolism, and differentiation state.

In addition to all previously described asexual and sexual stages (Wilke et al., 2018), we identified a stage we termed “late-stage macrogamonts” in which WFBs organized in a circular pattern at the perimeter of the cell. A similar process occurs during formation of the oocyst wall in the related coccidian *Eimeria* (Belli et al., 2009, Mai et al., 2009), suggesting that these “late-stage macrogamonts” may represent zygotes of *C. parvum* that form in ALI but not conventional cell cultures. Mature oocysts appeared by day 3 post-infection and were present in cyclical waves, suggesting they do not accumulate as a mature end stage, but rather excyst to reinitiate infection. Oocysts exhibited features of the mature oocyst wall including lectin staining, were bleach-resistant, and were infectious to naive monolayers and to mice, leading to robust oocyst shedding.

The growth of *C. parvum* in ALI transwells has several advantages over alternative in vitro methods described previously. For example, ALI transwell cultures grown in microtiter format can be easily scaled (e.g., multiples of 12 transwells can be plated simultaneously) to test multiple conditions in parallel. The availability of long-term growth in ALI monolayers also facilitates genetic approaches in *C. Parvum*, as demonstrated using CRISPR/Cas9, to generate transgenic parasites *in vitro* that were then further amplified in the mouse. The ability of *C. parvum* to complete the entire life cycle in ALI cultures was also used to perform genetic crosses in vitro, something currently not possible with other apicomplexan parasites. To fully capitalize on this development, it would be advantageous to develop methods for serial propagation, for the generation of single cell clones, and for regulatable systems to study essential genes. Such barriers have rapidly eroded where efforts have been applied in other parasitic parasites, and they are likely to do so here as well.

The segregation pattern of independent markers on different chromosomes observed following in vitro genetic crosses of *C. parvum* is consistent with the fusion of haploid gametes followed by independent segregation of chromosomes, as predicted by Mendelian genetics. A survey of the highly reduced *C. parvum* genome indicates that it retains nearly all of the conserved genes within the meiotic tool box (Schurko and Logsdon, 2008) (Table S1). The ability to recover all four progeny from each meiotic event could facilitate future studies to understand the meiotic division processes used by apicomplexans. Additionally, genetic mapping and linkage analysis should be useful for forwarding genetic analysis of complex traits in *C. parvum*. In this regard, the ability of *C. parvum* to complete its entire life cycle and undergo meiosis within 3 days within a single in vitro culture offers considerable advantages over other parasitic systems that must rely on multiple invertebrate and/or vertebrate hosts to perform such genetic crosses.

## STAR★Methods

### Key Resources Table

**Table:** 

REAGENT OR RESOURCE	SOURCE	IDENTIFIER
**Antibodies**		

Rabbit polyclonal anti-Chromogranin A	Abcam	Cat# ab15160
Mouse monoclonal anti-Villin [3E5G11] – N-terminal	Abcam	Cat# ab201989
Rabbit monoclonal anti-Ki67 [SP6]	Abcam	Cat# ab16667
Rabbit polyclonal anti-Mucin 2 (H-300)	Santa Cruz Biotechnology	Cat# sc-15334
Goat polyclonal anti-Lysozyme C antibody (C-19)	Santa Cruz Biotechnology	Cat# sc-27958
Fluorescein-conjugated mouse monoclonal Crypt-a-Glo^™^	Waterborne, Inc.	Cat# A400FLR-1X
Mouse monoclonal 1B5	Wilke et al., 2018	N/A
Mouse monoclonal 1A5	Wilke et al., 2018	N/A
Mouse monoclonal 4D8	Wilke et al., 2018	N/A
Mouse monoclonal 1E12	Wilke et al., 2018	N/A
Rabbit polyclonal pan Cp	This paper	N/A
Rabbit polyclonal anti-GFP	Thermo Fisher Scientific	Cat# A11122
Rat monoclonal anti-mCherry (16D7)	Thermo Fisher Scientific	Cat# M11217
Alexa Fluor 488 goat anti-mouse IgG (H+L)	Thermo Fisher Scientific	Cat# A11029
Alexa Fluor 488 goat anti-rabbit IgG (H+L)	Thermo Fisher Scientific	Cat# A11034
Alexa Fluor 568 goat anti-rabbit IgG (H+L)	Thermo Fisher Scientific	Cat# A11011
Alexa Fluor 568 goat anti-rat IgG (H+L)	Thermo Fisher Scientific	Cat# A11077
Alexa Fluor 568 donkey anti-goat IgG (H+L)	Thermo Fisher Scientific	Cat# A11057
Alexa Fluor 647 goat anti-mouse IgG (H+L)	Thermo Fisher Scientific	Cat# A21235
FITC-conjugated Maclura pomifera lectin (Osage Orange) – MPA	EY Laboratories, Inc.	Cat# F-3901-1
FITC-conjugated Ulex europaeus lectin (Gorse, Furze) – UEA-I	EY Laboratories, Inc.	Cat# F-2201-2

**Bacterial and Virus Strains**		

NEB 5-alpha Competent *E. coli* (High Efficiency)	New England Biolabs	Cat# C2987H

**Chemicals, Peptides, and Recombinant Proteins**		

Y-27632 dihydrochloride ROCK inhibitor	Tocris Bioscience	Cat# 1254
BD Matrigel^™^ Basement Membrane Matrix	BD Biosciences	Cat# 356234
Glutaraldehyde	Polysciences, Inc.	Cat# 01909
Osmium tetroxide	Polysciences, Inc.	Cat# 0972B
Formaldehyde (methanol-free), Ultrapure EM Grade	Polysciences, Inc.	Cat# 04018-1
Uranyl Acetate	Ted Pella, Inc.	Cat# 19481
Eponate 12 resin kit	Ted Pella, Inc.	Cat# 18012
Lead nitrate	Electron Microscopy Sciences	Cat# 17900
Sodium citrate	Sigma	Cat# S-4641
Paromomycin sulfate salt	Sigma	Cat# P9297
Sodium taurocholate hydrate	Sigma	Cat# 86339
Bovine serum albumin	Sigma	Cat# A7030
2-mercaptoethanol	Sigma	Cat# M6250
Poly-L-Lysine solution (0.01%)	Sigma	Cat# P4707-50mL
TAT-CRE Recombinase	Millipore Sigma	Cat# SCR508
Triton X-100	Thermo Fisher Scientific	Cat# BP151–100
ProLong^™^ Diamond Antifade Mountant	Thermo Fisher Scientific	Cat# P36970
Hoechst 33342	Thermo Fischer Scientific	Cat# C10637
JAG-1 peptide	AnaSpec	Cat# AS-61298
GelRed nucleic acid gel stain	Biotium	Cat# 41003-1

**Critical Commercial Assays**		

Click-iT Plus EdU Alexa Fluor 488 Imaging kit	Thermo Fisher Scientific	Cat# C10637
Click-iT EdU Alexa Fluor 594 Imaging kit	Thermo Fisher Scientific	Cat# C10339
QIAamp DNA Mini kit	QIAGEN	Cat# 51306
QIAamp DNA Stool Mini kit	QIAGEN	Cat# 51504
RNeasy Mini kit	QIAGEN	Cat# 74104
QIAshredder	QIAGEN	Cat# 79654
DNA-free DNA Removal kit	Thermo Fisher Scientific	Cat# AM1906
TB Green^™^ Advantage® qPCR premix	Takara Bio, Inc.	Cat# 639676
Gibson Assembly Cloning kit	New England Biosciences	Cat# E5510S
Q5 Site-directed Mutagenesis kit	New England Biosciences	Cat# E0554S
Q5 Hot Start High-Fidelity 2X master mix	New England Biosciences	Cat# M0494S
SF Cell Line 4D-Nucleofector X Kit L	Lonza	Cat# V4XC-2024
Nano-Glo Luciferase Assay kit	Promega	Cat# N1120

**Deposited Data**		

Raw and analyzed RNA-seq data	This paper	GEO: GSE124815

**Experimental Models: Cell Lines**		

Mouse: NIH/3T3	ATCC	CRL-1658
Mouse: L-WRN	ATCC	CRL-3276
Mouse: Ileal epithelial stem cells from C57BL/6	Laboratory of Thaddeus Stappenbeck	N/A
Human: HCT-8	ATCC	CCL-244

**Experimental Models: Organisms/Strains**		

Mouse: Nod *scid* gamma (NSG^™^)	Jackson Laboratories	Cat# 005557
Mouse: Ifngr1 KO (C57BL/6 background)	Jackson Laboratories	Cat# 003288
Mouse: Atoh1 flox	Jackson Laboratories	Cat# 008681
*Cryptosporidium parvum* AUCP-1 isolate	Laboratories of Mark Kuhlenschmidt or William Witola	N/A

**Oligonucleotides**		

Primer: *C. parvum* GAPDH Forward: CGGATGGCCATACCTGTGAG	This paper	N/A
Primer: *C. parvum* GAPDH Reverse: GAAGATGCGCTGGGAACAAC	This paper	N/A
Primer: mouse GAPDH Forward: GCCATGAGTGGACCCTTCTT	This paper	N/A
Primer: mouse GAPDH Reverse: GAAAACACGGGGGCAATGAG	This paper	N/A
Primer: Atoh1-P1 GCGCAGCGCCTTCAGCAACC	Bjerknes et al., 2012	N/A
Primer: Atoh1-P2 GACCTGTCGCCTTCGCTGCC	Bjerknes et al., 2012	N/A
Primer: Atoh1-P3 GCGCGCTAGGAAGGGCATTTGG	Bjerknes et al., 2012	N/A

**Recombinant DNA**		

Plasmid: TK-GFP-Nluc-P2A-neo-TK	This paper	N/A
Plasmid: UPRT-mCh-Nluc-P2A-neo-UPRT	This paper	N/A
Plasmid: pACT1:Cas9-GFP, U6:sgTK	This paper; Addgene	Cat# 122852
Plasmid: pACT1:Cas9-GFP, U6:sgUPRT	This paper; Addgene	Cat# 122853
Plasmid: pUC19 vector	New England Biolabs, Inc.	Cat# N3041S

**Software and Algorithms**		

Partek Flow	Partek, Inc.	http://www.partek.com/partek-flow/
Ingenuity Pathway Analysis	QIAGEN	https://www.qiagenbioinformatics.com/products/ingenuity-pathway-analysis/
SnapGene	GSL Biotech	https://www.snapgene.com/
Eukaryotic Pathogen CRISPR guide RNA/DNA design tool	EuPaGDT	http://grna.ctegd.uga.edu
FIJI (ImageJ)	Schindelin et al., 2012	https://fiji.sc/
GraphPad Prism	GraphPad Software	https://www.graphpad.com/scientific-software/prism/

### Contact for Reagent and Resource Sharing

Further information and requests for resources and reagents should be directed to and will be fulfilled by the Lead Contact, L. David Sibley (sibley@wustl.edu).

### Experimental Model and Subject Details

#### *Cryptosporidium* Strain

*C. parvum* oocysts (AUCP-1 isolate) were maintained by repeated passage in male Holstein calves and purified from fecal material after sieve filtration, Sheather’s sugar flotation, and discontinuous sucrose density gradient centrifugation as previously described (Kuhlenschmidt et al., 2015). All calf procedures were approved by the Institutional Animal Care and Use Committee (IACUC) at the University of Illinois Urbana-Champaign. Purified oocysts were stored at 4°C in PBS + 50 mM Tris-10 mM EDTA, pH 7.2 for up to six months before use.

#### Cell Lines

All cell lines were cultured at 37°C in a 5% CO_2_ incubator under normal atmospheric oxygen conditions. Primary ileal intestinal epithelial stem cells (IECs) isolated from 8-10-week-old female C57BL/6 mice (Stappenbeck laboratory, Washington University School of Medicine) were expanded and maintained as 3D spheroid cultures in Matrigel (BD Biosciences) and 50% L-WRN conditioned medium (CM) containing 10 μM Y-27632 ROCK inhibitor (Tocris Bioscience), as described previously (Miyoshi et al., 2012). L-WRN-CM was quality controlled form batch to batch using recently defined methods (VanDussen et al., 2019). The medium was changed every 2 days, and the cells were passaged every 3 days in a 1:6 split. IEC lines were determined to be mycoplasma-negative using the e-Myco plus kit (Intron Biotechnology). For all experiments in this study, IECs were used between passages 4 and 26.

NIH/3T3 embryonic mouse fibroblast cells (ATCC, CRL-1658^™^) were maintained in Dulbecco’s Modified Eagle’s Medium (DMEM high glucose; Sigma D6429) with 10% fetal bovine serum (Sigma) and 1X penicillin/streptomycin (Sigma). Cells were passaged every 3 days in a 1:5 split.

HCT-8 human colorectal adenocarcinoma cells from a male patient (ATCC, CCL-244) were maintained in RPMI 1640 ATCC Modification medium (Thermo Fisher Scientific A1409101) supplemented with 10% fetal bovine serum (Sigma). Cell were passaged twice a week at a 1:5 – 1:20 split.

#### Mouse Lines

For the bleach, ALI infectivity assays, female 8- to 10-week-old Ifngr1^-/-^ mice (C57BL/6 genetic background) were purchased directly from Jackson Laboratories (#003288) and housed separately for the duration of the experiment to avoid cross-infection. For amplification of transgenic *C. parvum* parasites, male and female 7- to 14-week-old Ifngr1^-/-^ mice and male and female 12- to 17-week-old Nod *scid* gamma mice (NSG^™^, Jackson Laboratories #005557) were bred in house at Washington University School of Medicine and co-housed based on sex. Mice were reared in a specific-pathogen-free facility on a 12:12 light-dark cycle and received irradiated laboratory rodent chow (Purina 5053) and water *ad libitum*. For amplification of transgenic parasites, water was replaced with filtered tap water containing 16 g/L paromomycin sulfate salt (Sigma). Animals that lost more than 20% of their body weight or became non-ambulatory during the course of infection were humanely euthanized in a SMARTBOX Auto CO_2_ euthanasia chamber. All mouse studies were approved by the Institutional Animal Studies Committee at the School of Medicine, Washington University in St. Louis.

### Method Details

#### Generating the Air-Liquid Interface Mouse Intestinal Epithelial Cell Culture System

##### Irradiating 3T3 Fibroblast Cells

Mouse fibroblast cells (NIH/3T3; CRL-1658^™^ ATCC) were trypsinized, suspended in growth medium, and irradiated at 3,000 rads using the Small Animal Radiation Research Platform (SARRP, Xstrahl). After cell viability was assessed with Trypan Blue staining (Thermo Fisher), irradiated cells were quantified, aliquoted in freezing medium (growth medium with 30% FBS and 5% DMSO) and stored at -80°C for short-term use (weeks) or in liquid nitrogen for long-term use (months).

##### Seeding Irradiated 3T3 Feeder Cell Layer on Transwells

Transwells (polyester membrane, 0.4 μm pore; Corning Costar) were coated with Matrigel (Corning) diluted 1:10 in cold PBS, then incubated at 37°C for 15-20 min. Excess Matrigel solution was aspirated immediately before adding irradiated 3T3 (i3T3) cells, which were thawed, resuspended in growth medium, and seeded onto transwells at 8x10^4^ cells/ transwell. Growth medium was added to the top and bottom of the transwell and incubated at 37 °C for approximately 24 hr before seeding the mIEC spheroids.

##### Seeding the Mouse Intestinal Epithelial Cell Monolayers and Creating Airliquid Interface

Mouse ileal spheroids from 3-day-old stem cell cultures were recovered from Matrigel and dissociated with trypsin as described previously (Moon et al., 2014). Cells were quantified, suspended in 50% CM with 10 μM Y-27632, and plated onto i3T3 monolayers at 5x10^4^ cells/ transwell. 3T3 growth medium was replaced with 50% CM with 10 μM Y-27632 in both the top and bottom compartments and replenished every other day. After 7 days, the medium in the top compartment was removed to create the air-liquid interface (ALI). Medium in the bottom compartment of the transwell continued to be changed every other day. Liquid/mucus that appeared in top compartment was removed every other day.

##### Generating Mouse Atoh1^-/-^ Knockout Spheroid Lines

Spheroids were established from the jejunum of Atoh1 flox mice (Jackson Laboratory Stock # 008681) (Shroyer et al., 2007). For Tat-Cre mediated recombination, 5 μM Tat-Cre recombinase (Millipore Sigma) was added to a single-cell suspension of spheroid epithelial cells in 50% L-WRN CM with 10 μM ROCK Inhibitor (Y-27632; Tocris Bioscience) overnight. The following day, the cells were pelleted, plated in Matrigel containing 1 μM Jag1 peptide (AnaSpec), and cultured as described above. Single spheroids were selected, expanded, and then genotyped for recombination of the flox alleles by PCR with the following primers (Bjerknes et al., 2012): Atoh1-P1 5’-GCGCAGCGCCTTCAGCAACC-3’; Atoh1-P2 5’-GACCTGTCGCCTTCGCTGCC-3’; Atoh1-P3 5’-GCGCGCTAGGAAGGGCATTTGG-3’.

#### RNA Sequencing of Uninfected ALI vs Non-ALI Monolayers

##### RNA Collection and Purification

RNA was harvested from uninfected transwells seven days post IEC seeding on the day of top medium removal (ALI D0), ten days post IEC seeding with continuous top medium (non-ALI), and ten days post IEC seeding with three days of ALI (ALI D3). To collect RNA, transwells were scraped in RLT buffer (QIAGEN) + 10 μl/ml 2-mercaptoethanol, transferred to a Qiashredder column (QIAGEN), spun at max speed for two mins, then stored at -80 °C until further processing. Two transwells were combined per column with two replicates at each timepoint from two independent experiments for a total of 12 samples. Concurrently, RNA from stem-cell spheroids were harvested 3 days post seeding, one well per Qiashredder column, two replicates per experiment from two independent experiments for a total of four spheroid samples. RNA was extracted and purified from all samples using the RNeasy Mini kit (QIAGEN) then treated with the DNase DNeasy kit (Thermo Fisher Scientific) to remove residual DNA.

##### Library Preparation and Sequencing

Total RNA was submitted to the Genome Access Technology Center (Washington University School of Medicine) for library prep and sequencing. RNA integrity was determined using an Agilent Bioanalyzer and ribosomal RNA was removed using a Ribo-ZERO kit (Illumina-EpiCentre). mRNA was then fragmented and reverse-transcribed using the SuperScript III RT enzyme (Life Technologies) and random hexamers. A second strand reaction was performed to yield ds-cDNA, which was then blunt-ended and ligated to Illumina sequencing adapters. Ligated fragments were amplified for 12 cycles using primers incorporating unique index tags. Two biological replicates per sample (eight samples total) were multiplexed for single-end, 50 bp reads on a single lane of an Illumina HiSeq3000. To increase coverage and sample size, the other two biological replicates per sample were independently processed and sequenced with identical parameters to the first set.

Demultiplexed fastq files were imported into Partek Flow (Partek, Inc.) with a mean of 43,115,081 reads per sample (range: 31,092,799 – 50,130,171 reads). Five bases were trimmed from the 5′ end of all reads to increase the average quality score across samples from 38.92 to 39.45. Trimmed reads were then mapped to the *Mus musculus* mm10 genome build (NCBI GenBank assembly ID GCA_000001635) using the STAR aligner with default parameters (Dobin et al., 2013), and the number of reads per gene was quantified based on the mouse Ensembl Transcripts release 90. On average, 88.9 ± 6.1% (mean ± S.D.) of reads per sample were mapped to the genome with an average coverage of 577.76 reads per gene (13,889 genes total).

##### Analysis of RNA-Seq Data

For differential expression analyses, we first normalized gene expression values by dividing the number of reads per gene by the total number of reads per sample to obtain counts per million (CPM) values. These normalized expression values were used in a principal component analysis to visualize the variation in expression differences between all replicates and sample types. Pair-wise comparisons of sample types (4 replicates each) were performed using the Partek gene-specific analysis (GSA) algorithm, which is a multimodel approach that determines the best response distribution (e.g. lognormal, Poisson, or negative binomial) for each gene based on Akaike information criteria (AICc) model selection rather than fitting all data to one distribution. Genes were not included in the analysis if their mean CPM value over all samples was less than 1. Genes were considered significantly differentially expressed if the FDR-corrected *P value* was less than 0.05 and the absolute fold change was greater than 2.

For molecular pathway analyses of day 3 ALI and non-ALI samples, the expression fold change and FDR-corrected *P value* for all genes from Partek Flow were imported into Ingenuity Pathway Analysis (QIAGEN). A core analysis was performed using the Ingenuity Knowledge Database to identify canonical pathways and gene networks that changed significantly (Z-score < 0.05) under ALI growth conditions. A separate core analysis was also performed using as input only genes that were significantly differentially expressed between day 3 ALI and non-ALI (FDR-corrected *P value* < 0.05, absolute fold change > 2).

#### *C. parvum* Oocyst Preparation, Excystation, and Infection of ALI Transwells

Before infection, purified *C. parvum* oocysts were treated with a 40% bleach solution (commercial laundry bleach containing 8.25% sodium hypochlorite) diluted in Dulbecco’s Phosphate Buffered Saline (DPBS; Corning Cellgro) for 10 min on ice. Oocysts were then washed 4 times in DPBS containing 1% (wt/vol) bovine serum albumin (BSA; Sigma) before resuspending to a final concentration of 1x10^8^ oocyst/ml in DPBS with 1% BSA. For some experiments, oocysts were excysted prior to infection by incubating the oocysts with 0.75% sodium taurocholate (w/v; Sigma) in DPBS at 37°C for 60 min. As indicated, excysted oocysts were filtered through a membrane with 1 μm pore size (Whatman, VWR International) to remove unexcysted oocysts from sporozoites. Sporozoites were spun down at 1,250 x g for 3 min and then resuspended in 50% CM prior to adding to ALI monolayers. Oocysts or filtered sporozoites were added to monolayers in 30 μl of 50% CM three days post top medium removal. After 3 hr, monolayers were washed twice with DPBS to remove extracellular parasites and re-establish the air-liquid interface.

#### Measuring *C. parvum* Growth and Host Cell Viability by qPCR

To monitor infection by quantitative PCR, DNA was collected and purified from infected transwells using the QIAamp DNA Mini kit (Qiagen). Briefly, 100 μl Buffer ATL (provided by kit) was added to monolayer, then cells were scraped into buffer using a blunt pipette tip. Lysed cells were incubated in Buffer ATL and proteinase K (both reagents provided by kit) in a 56°C water bath for 3-24 hr before column purification. Purified DNA was eluted in 100 ul Buffer AE then diluted 1:10 with H_2_O. Two μl of the diluted DNA was used as template in a qPCR reaction with TB Green^™^ Advantage® qPCR premix (Takara, Clontech). Primer sequences targeting C. parvum GAPDH were as follows: forward primer 5’-CGGATGGCCATACCTGTGAG-3’ and reverse primer 5’-GAAGATGCGCTGGGAACAAC-3’. A standard curve for *C. parvum* genomic DNA was generated by purifying DNA from a known number of oocysts and creating a dilution series. Primer sequences targeting mouse GAPDH were as follows: forward primer 5’-GCCATGAGTGGACCCTTCTT-3’ and reverse primer 5’-GAAAACACGGGGGCAATGAG-3’. A standard curve for mouse genomic DNA was generated by purifying DNA from a known number of mouse ileal stem cells and creating a dilution series. Reactions were performed on a QuantStudio 3 Real-Time PCR System (Thermo Fisher) with the following amplification conditions: priming at 95°C for 2 min then 40 cycles of denaturing at 95°C for 10 sec, annealing at 60°C for 20 sec, extension at 72°C for 30 sec, followed by a melt curve analysis to detect non-specific amplification. Genomic DNA equivalents in each sample were determined by the QuantStudio Design & Analysis software based on the standard curve samples present on each plate.

#### Bleaching ALI Monolayers for Microscopy, Animal Infection and *In Vitro* Passage

ALI monolayers were infected on day 3 post top medium removal with filtered sporozoites. After 2 hr, monolayers were washed twice with DPBS. On specified days post infection, monolayers were scraped into cold 40% bleach diluted in DPBS, combined into a single Eppendorf tube, and bleached on ice for 10-15 min before spinning down at maximum speed for 2 min. The bleach solution was removed, and the pellet was washed 6 times in 1 ml cold DPBS, repeating the centrifugation step each time, before resuspending in the desired volume of cold DPBS.

#### Generating the Pan Cp Polyclonal Antiserum

Antigen for the pan Cp polyclonal antiserum was generated by excysting 8 x 10^8^ bleached oocysts in 0.75% sodium taurocholate at 37°C for 1 hr; excysted oocysts were then freeze-thawed 6 times (3 min on dry ice mixed with ethanol, then 3 min at 37°C). Sample was sent to Covance (Princeton, N.J, USA) for immunization. One rabbit was injected subcutaneously with 250 mg antigen with Freund’s Complete Adjuvant (FCA) then boosted three times at 21-day intervals with 125 mg antigen in Freund’s Incomplete Adjuvant (FIA). All immunofluorescent staining experiments used the terminal bleed pan Cp antiserum at a 1:1,000 dilution.

#### Immunofluorescence Microscopy

Transwells were moved to a new 24-well plate with DPBS in the bottom chamber. Monolayers were fixed by adding 100 μl 4% formaldehyde (Polysciences) for 10-15 min. Cells were washed twice with DPBS and then permeabilized and blocked with DPBS containing 1% BSA and 0.1% Triton X-100 (Sigma) for 20 min. Primary antibodies were diluted in blocking buffer for staining: 1B5 and 1A5 (purified mouse mAbs) were used at 1:500 (Wilke et al., 2018), pan Cp (rabbit pAb) was used at 1:1000, Crypt-a-glo^™^ (mouse mAb, Waterborne, Inc) was used at 1 drop per 2 transwells, and 4D8 (hybridoma supernatant) was used at 1:5 (Wilke et al., 2018).Cells were incubated with primary antibodies for 60 min at room temperature, washed twice with PBS, then incubated for 60 min at room in secondary antibodies conjugated to Alexa Fluor dyes (Thermo Fisher Scientific) diluted 1:1,000 in blocking buffer. Nuclear DNA was stained with Hoechst (Thermo Fisher Scientific) diluted 1:1,000 in blocking buffer for 10 mins at room temperature, then the membrane was cut out of the transwell insert using a scalpel and mounted on a glass slide with Prolong Diamond Antifade Mountant (Thermo Fisher Scientific).

For lectin staining of bleached ALI monolayers, bleached material was pipetted onto 0.01% poly-L-lysine (Sigma) treated coverslips in a 24-well plate (150-200 μl total volume per well), allowed to settle for 30 min, then fixed and permeabilized as described above. Coverslips were stained with specified lectin for 30 minutes, followed by pan Cp staining for 60 min and secondary antibody staining for 60 min, all at room temperature. Lectins used were FITC-conjugated *Maclura pomifera* (MPA) lectin (E Y Laboratories, F-3901-1) and FITC-conjugated *Ulex europaeus* (UEA-1) lectin (E Y Laboratories, F-2201-2), both used at 100 μg/ml.

For EdU staining of replicating DNA, 10 μM EdU was added to medium in the bottom chamber of the transwell. After a defined time period, the transwell was fixed with 4% formaldehyde and permeabilized as described above. EdU was labeled with the Click-iT Plus EdU Alexa Fluor 488 (Thermo Fisher Scientific, C10637) or 594 (Thermo Fisher Scientific, C10339) Imaging Kits. Primary and secondary antibody staining were done after EdU labeling.

Imaging was performed on a Zeiss Axioskop Mot Plus fluorescence microscope equipped with a 100X, 1.4 N.A. Zeiss Plan Apochromat oil objective and an AxioCam MRm monochrome digital camera. Images were acquired using Axiovision software (Carl Zeiss Inc.) and manipulated in ImageJ or Photoshop.

#### Sectioning and Staining Transwells for Histology

Transwells were treated with 4% formaldehyde or 4% paraformaldehyde in both top and bottom chambers for 20 min at room temperature, washed three times in 70% ethanol, then incubated for 20 min in 70% ethanol (top and bottom chambers). The transwell membranes were cut from the insert using a scalpel and embedded in 1% agar and then processed for paraffin embedding. For hematoxylin and eosin (H&E) staining and immunohistochemistry, five μm transverse sections were cut and processed for staining following standard procedures of the Digestive Disease Research Core Center (DDRC, Washington University in St. Louis). Sections were imaged using a Zeiss Observer.D1 inverted wide-field fluorescence microscope with Axiocam 503 dual B/W and color camera.

For immunostaining, slides were deparaffinized, and antigen retrieval was performed with Trilogy. Slides were then stained as described above using the following antibodies: mouse anti-villin (1:1000, Abcam) with goat anti-mouse IgG Alexa Fluor 488 (Thermo Fisher Scientific); rabbit anti-Chromogranin A antibody (1:1000, Abcam), rabbit anti-mucin 2 (1:200, SantaCruz), and rabbit anti-Ki-67 (1:400, Abcam) with goat anti-rabbit IgG Alexa Fluor 568 (Thermo Fisher Scientific); goat anti-lysozyme (1:100, SantaCruz) with donkey anti-goat IgG Alexa Fluor 568 (Thermo Fisher Scientific); and Hoechst for nuclear staining. Images were taken using a 40x oil immersion objective (N.A. 1.30) on a Zeiss Axioskop 2 equipped for epifluorescence.

#### Transmission Electron Microscopy

For ultrastructural analyses, ALI cultures were fixed in a freshly prepared mixture of 1% glutaraldehyde (Polysciences, Inc) and 1% osmium tetroxide (Polysciences, Inc.) in 50 mM phosphate buffer at 4°C for 30 min. Samples were then rinsed multiple times in cold dH_2_0 prior to en bloc staining with 1% aqueous uranyl acetate (Ted Pella Inc.) at 4°C for 3 hr. Transwell membranes were removed from insert using a scalpel. Following several rinses in dH_2_0, samples were dehydrated in a graded series of ethanol and embedded in Eponate 12 resin (Ted Pella, Inc.). Sections of 95 nm were cut with a Leica Ultracut UCT ultramicrotome (Leica Microsystems, Inc.), stained with uranyl acetate and lead citrate, and viewed on a JEOL 1200 EX transmission electron microscope (JEOL USA, Inc.) equipped with an AMT 8-megapixel digital camera and AMT Image Capture Engine V602 software (Advanced Microscopy Techniques).

#### Testing Infectivity of Bleached ALI Cultures in Ifngr1^-/-^ Mice

Female 8- to 10-week-old Ifngr1^-/-^ mice from Jackson Laboratories were orally gavaged with 200 μl of bleached ALI material (equivalent to 4-5 transwells) from either day 1 (n = 4 mice) or day 3 (n = 4 mice) post-infection cultures. After gavaging, mice were housed separately for the duration of the experiment to avoid cross-infection. One mouse infected with bleached, day 1 ALI culture material was sacrificed on day 30 post-infection and one mouse infected with bleached, day 3 ALI culture material was sacrificed on day 9 post-infection to collect the small intestine for histology as described above. Mouse pellets were collected every 2-3 days, and the mice were monitored for signs of sickness. Mouse pellets were kept at -80°C until they were processed for DNA extraction, which was performed using the QIAamp DNA Stool Kit (QIAGEN). Pellets were moved to Lysing Matrix E 2 ml tubes (MP Biomedicals) and 1.4 ml ASL Buffer (from kit) was added. Samples were homogenized using the FastPrep-24^™^ 5G High-Speed Homogenizer, then processed according to the kit’s directions. qPCR was used to quantify the number of *C. parvum* genomic DNA equivalents present in the sample using the *C. parvum* GAPDH primers and cycling protocols as described above.

#### Cloning of *C. parvum* CRISPR/Cas9 and Targeting Plasmids

To generate a CRISPR/Cas9 plasmid for use in *C. parvum*, we used restriction cloning with SacI to insert the *C. parvum* U6 gene into a pUC19 vector (New England Biosciences) to create pUC19-CpU6. We then inserted the *C. parvum* actin promoter (984 bp upstream of cgd5_3160) upstream of Cas9-NLS-GFP (Jinek et al., 2013, Shen et al., 2014), followed by the *C. parvum* actin 3’ UTR region (562 bp downstream of cgd5_3160) into puc19-CpU6 by Gibson assembly (New England Biosciences) to create pACT1:Cas9-GFP. This plasmid (pACT1:Cas9-GFP) was further modified by inserting the thymidine kinase (TK, cgd5_4440) guide RNA (sgRNA) and the tracrRNA amplified from the Aldolase_Cas9 plasmid (Vinayak et al., 2015) downstream of the CpU6 promoter using Gibson assembly to create pACT1:Cas9-GFP, U6:sgTK (Addgene 122852). The plasmid pACT1:Cas9-GFP, U6:sgUPRT (Addgene 122853) was generated by replacing the TK sgRNA with a sgRNA targeting the *C. parvum* uracil phosphoribosyltransferase gene (*uprt*, cgd1_1900) using Q5 site-directed mutagenesis (New England Biosciences). The UPRT sgRNA was designed using the Eukaryotic Pathogen CRISPR guide RNA/DNA Design tool (http://grna.ctegd.uga.edu) searching against the *C. parvum* IowaII CryptoDB-28 genome to avoid off-target effects.

To generate *tk* knockout mutants that express GFP, we made a TK-GFP-Nluc-P2A-neo-TK targeting plasmid by first deleting the Cas9 and NLS sequences from the pACT1:Cas9-GFP plasmid described above using Q5 mutagenesis. This generated a GFP construct expressed under the *C. parvum* actin promoter that was then inserted between the 5’ *tk* homology flank and the enolase-Nluc-neo^R^ reporter in the previously published *C. parvum tk* targeting plasmid (Vinayak et al., 2015) by Gibson assembly. A P2A skip peptide (Doherty et al., 1999, Tang et al., 2016) was inserted between the nanoluciferase and neomycin resistance CDS by Q5 mutagenesis, then the entire construct was subcloned into a high copy-number vector (pUC19) to increase plasmid yield. To make *uprt* knockout mutants that express mCherry, we edited the *tk* targeting construct by swapping the GFP CDS with mCherry from pLoxP-DHFR-TS-mCherry (Behnke et al., 2015) using Gibson assembly and replacing the *tk* homologous flanks with *uprt* homologous flanks (800 bp upstream and 913 bp downstream of cgd1_1900) by restriction cloning with XmaI and PstI (New England Biosciences).

#### Transfection of *C. parvum* Sporozoites and Selection in ALI

Oocysts were excysted as described above (1.25 x 10^7^ oocysts per cuvette), then sporozoites were pelleted by centrifugation and resuspended in SF buffer (Lonza) containing 50 ug targeting plasmid and 30 ug CRISPR/Cas9 plasmid for a total volume of 100 μl. Sporozoites were then transferred to a 100 μl cuvette (Lonza) and electroporated on an AMAXA 4D-Nucleofector System (Lonza) using program EH100. Electroporated sporozoites were transferred to 50% L-WRN conditioned medium then added to the top of eight ALI transwells per cuvette (30 μl volume, approx. 6.25 x 10^6^ sporozoites per transwell). At 8 hpi, top media was removed, transwells were washed 2X on top with sterile DPBS, and bottom media was replaced with 50% CM + 20 mM paromomycin (Sigma) with 1M NaOH added to bring the pH to ∼7.4. Bottom media with drug was replaced every two days.

#### Amplifying Transgenic Parasites in Immunodeficient Mice

Transgenic parasites were drug-selected for 5 – 7 days in ALI transwells with paromomycin in the bottom medium before transferring to mice for amplification. To prepare samples for gavage, 1 – 2 transwells per mouse were scraped in DPBS, combined in a single Eppendorf tube, and syringe-lysed five times with a 20-gauge needle, then five times with a 23-gauge needle. Sample volume was brought up to 200 μl per mouse in DPBS and orally gavaged into 3 – 4 Infgr1^-/-^ mice per transgenic *C. parvum* line. Mice infected with same transgenic parasite strain were co-housed but separated by sex. All mice received drinking water with 16 g/L paromomycin for the entirety of the experiment. Paromomycin dose was based on published protocols for selecting transgenic *C. parvum* strains in vivo (Pawlowic et al., 2017, Vinayak et al., 2015). Fecal pellets were collected in microcentrifuge tubes every 3 days for qPCR (stored at -80°C) and luciferase assays (4°C) and extra fecal pellets were collected and stored at 4°C starting 6 dpi. Mice were euthanized by CO_2_ asphyxiation once they became overtly ill, immobile, or lost 20% of their body weight.

A second round of amplification was performed by orally gavaging 3 – 4 Nod *scid* gamma (NSG) mice per transgenic line with a fecal slurry from the infected Ifngr1^-/-^ mice. Fecal slurries were produced by grinding a single pellet (9 dpi) in DPBS, then centrifuging at low speed (200 rpm) for 10 mins to pellet large particulates. The supernatant was then diluted in DPBS to achieve a concentration of approx. 1 x 10^4^ oocysts per mouse based on the number of oocysts per mg feces for a separate pellet from the same mouse on the same day as measured by qPCR. Experimental setup and execution were the same as with Infgr1^-/-^ mice, including selection with 16 g/L paromomycin drinking water for the entirety of the experiment. Fecal pellets for oocyst purification were collected every day starting 12 dpi and stored at 4°C. For purification, fecal samples from all mice were pooled and oocysts extracted as previously described (Zhang et al., 2018). Purified oocysts were stored in PBS at 4°C and used within six months of extraction.

#### Luciferase Assay

All luciferase assays were performed with the Nano-Glo Luciferase Assay kit (Promega). For infected ALI cultures, transwells were incubated in 100 μl Nano-Glo Luciferase buffer (top compartment only) at 37°C for 15 mins. Cells were then scraped from the transwell membrane with a blunt pipette tip and transferred to a single well of a white 96-well plate (Greiner Bio-One). 100 μl of a 25:1 Nano-Glo Luciferase buffer to Nano-Glo Luciferase substrate mix was added to each well, and the plate was incubated for 3 min at room temperature. Luminescence values were read on a Cytation 3 Cell Imaging Multi-Mode Reader (BioTek). For mouse fecal pellets in 1.7 ml microcentrifuge tubes, pellets were ground with a pestle, then 3-mm glass beads (Fisher Scientific) and 1 ml fecal lysis buffer (50 mM Tris pH7.6, 2 mM DTT, 2 mM EDTA pH 8.0, 10% glycerol, 1% Triton X-100 prepared in water) (Pawlowic et al., 2017) were added to the tube. Tubes were incubated at 4°C for 30 mins, vortexed for 1 min, then spun at 16,000 x g for 1 min to pellet debris. 100 μl supernatant was added to two wells of a white 96-well plate, then substrate addition and luminescence reading was performed as above.

#### Fecal Pellet DNA Extraction, Oocyst Quantification and Insert PCR

For DNA extraction from fecal pellets, each frozen pellet was transferred to a 2 ml tube containing Lysing Matrix E beads (MP Biomedicals) and 1.4 ml ASL buffer (Qiagen), then homogenized using a FastPrep-24 5G benchtop homogenizer (MP Biomedicals). DNA was extracted after homogenization using the QIAamp DNA stool kit (Qiagen) according to manufacturer’s protocols. Oocyst numbers were quantified using qPCR with the *C. parvum* GAPDH primers as described above. To check for the successful insertion of the target sequence into either the *tk* or *uprt* locus, PCR was performed on 1 μl purified fecal DNA using Q5 Hot Start High-Fidelity 2X master mix (New England Biosciences) with primers listed in Table S2 at a final concentration of 500 nM each. PCR reactions were performed on a Veriti 96-well Thermal Cycler (Applied Biosystems) with the following cycling conditions: 98°C for 30 secs, followed by 35 cycles of 98°C for 15 secs, 64°C for 30 secs, and 72°C for 1.5 mins, with a final extension of 72°C for 2 mins. PCR products were run on 1.5% agarose gel containing GelRed (Biotium, diluted 1:10,000) and imaged on a ChemiDoc MP Imaging System (Bio-Rad).

#### Crossing Transgenic *C. parvum* in ALI and Quantifying Recombination Events

ALI transwells were infected three days post media removal with 5 x 10^4^ TK-GFP and 5 x 10^4^ UPRT-mCh oocysts (bleached and excysted as described above) in 30 μl 50% L-WRN conditioned medium added to the top compartment. Approximately six hpi, parasites were removed, and the top compartment was washed twice with sterile DPBS. At 1, 3 and 5 dpi, transwells were fixed in 4% formaldehyde and stained for immunohistochemistry as described above using polyclonal rabbit anti-GFP (Thermo Fisher Scientific) with goat anti-rabbit IgG Alexa Fluor 488 secondary and a monoclonal rat anti-mCherry 16D7 (Thermo Fisher Scientific) with goat anti-rat IgG Alexa Fluor 568. All antibodies were diluted 1:1000 in PBS + 0.1% Triton-X + 1% BSA. To quantify recombinant parasites expressing both GFP and mCherry, fields of mixed red and green parasites were imaged at random using a 63X oil immersion objective on a Zeiss Axioskop 2 equipped for epifluorescence. Red and green channels were then merged in ImageJ (Schindelin et al., 2012), and the number of yellow parasites per field was recorded. 13 – 26 fields were imaged from 1-2 transwells per experiment, and the experiment was performed twice.

To determine the frequency of oocysts produced from selfing versus crossing events, transwells were infected with TK-GFP and UPRT-mCh oocysts as described above. Three days p.i., 4 – 6 infected transwells were scraped and bleached as described above. Bleached material was used to infect two coverslips that had been plated with 4 x 10^5^ HCT-8 cells (ATCC CCL-244) 24 hrs prior to infection. Approximately 16 hpi with the bleached ALI material, HCT-8 coverslips were fixed and stained as described above with rabbit anti-GFP and goat anti-rabbit IgG Alexa Fluor 488; rat anti-mCherry and goat anti-rat IgG Alexa Fluor 568; and mouse monoclonal antibody 1E12 and goat anti-mouse IgG Alexa Fluor 647, which detects all intracellular stages of *C. parvum* (Wilke et al., 2018). Oocysts from the bleached material were plated at a low enough MOI that parasites originating from a selfing event versus a crossing event could be distinguished by the formation of parasite “clusters” containing parasites of all one color (selfing event) or with more than one color (crossing event). For clusters with parasites of more than one color, the number of parasites expressing GFP only (green), mCherry only (red), both reporters (yellow), or no reporter (“wild type” identified by staining with 1E12) per cluster was recorded. The number of each type of parasite was summed across all clusters per coverslip and expressed as a ratio of the total number of parasites counted. Data depicts results from two independent experiments.

### Quantification and Statistical Analysis

All statistical analyses were performed in GraphPad Prism 7 (GraphPad Software) unless otherwise specified. Non-parametric tests were used when available if data did not pass a Shapiro-Wilk test for normality or sample sizes were too low. A Mann Whitney U test was used when comparing the mean of two groups, while a Kruskal-Wallis test with a Dunn’s multiple comparisons test was used when comparing the means of one variable across three or more groups. When comparing the means of two or more groups across time, we used a two-way ANOVA corrected for multiple comparisons using either the Sidak method if means from two groups at the same time point were compared to each other or the Dunnett method if means for all time points were compared to the mean of the earliest time point. Statistical parameters for each experiment including statistical test used, technical replicates (n), independent biological replicates (N) and standard error are reported in the figure legends and associated method details.

#### Blinding and Inclusion/Exclusion

Samples were not blinded for analysis although they were repeated independently as stated in the text and figure legends. All samples were included in the analysis with one exception: In the RNA-seq studies, one of the spheroid samples had a high percentage of reads map to intergenic regions (38.03% of reads) indicative of DNA contamination, and hence it was removed from further analyses.

### Data and Software Availability

Raw RNA-seq reads and analyzed data generated in this study have been deposited in the Gene Expression Omnibus database under accession numbers GSM3554371-GSM3554385. The plasmids pACT1:Cas9-GFP, U6:sgUPRT and pACT1:Cas9-GFP, U6:sgTK have been submitted to Addgene and accession numbers are pending.

## Supplemental Information

Document S1. Figures S1–S7 and Table S1

Table S2. Primers Used in This Study, Related to STAR Methods

Document S2. Article plus Supplemental Information
