# Supplementary material for: A Stem-Cell-Derived Platform Enables Complete Cryptosporidium Development In Vitro and Genetic Tractability
Source: Cell Host Microbe. 2019 Jul 10;26(1):123–134.e8. doi: 10.1016/j.chom.2019.05.007 (PMC6617391; doi:10.1016/j.chom.2019.05.007)
Supplement: Document S1. Figures S1–S7 and Table S1 [file mmc1.pdf]

Cell Host & Microbe, Volume *26*

Supplemental Information

A Stem-Cell-Derived Platform Enables Complete *Cryptosporidium* Development *In Vitro* and Genetic Tractability

Georgia Wilke, Lisa J. Funkhouser-Jones, Yi Wang, Soumya Ravindran, Qiuling Wang, Wandy L. Beatty, Megan T. Baldridge, Kelli L. VanDussen, Bang Shen, Mark S. Kuhlenschmidt, Theresa B. Kuhlenschmidt, William H. Witola, Thaddeus S. Stappenbeck, and L. David Sibley

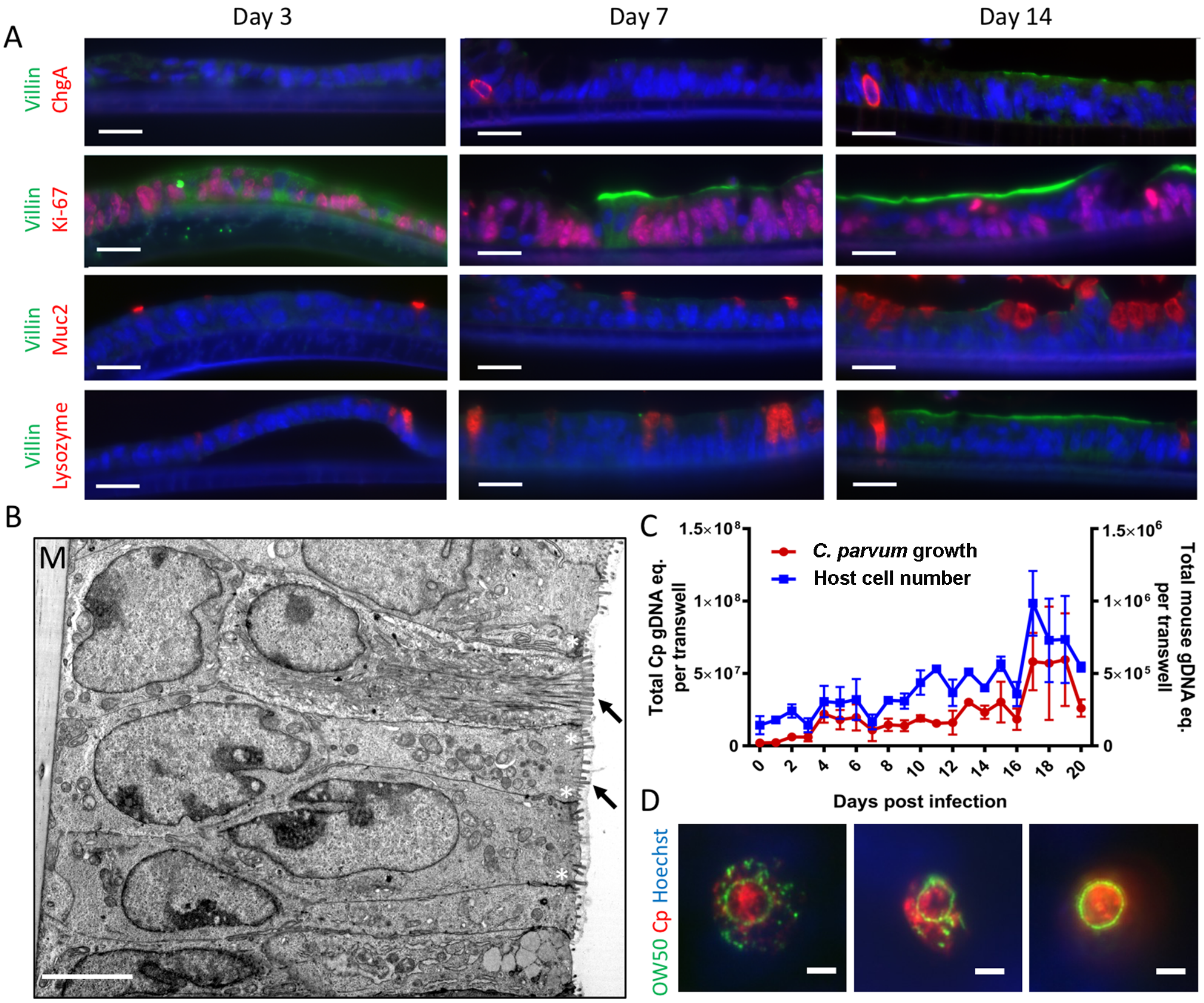

Figure S1 Development of ALI cultures for *C. parvum* cultivation. Related to Figure 1. (A) Immunofluorescence staining of intestinal epithelial cell lineages in ALI monolayers over time. Mouse intestinal epithelial cells (mIECs) propagated as spheroids were plated on transwells (2 x 10^5 cells per transwell) and cultivated under ALI conditions. At the indicated times post top medium removal, ALI cultures were fixed, embedded in paraffin, and sectioned. Immunofluorescence staining was performed on de-paraffinized sections with mouse anti-villin (stains the apical brush border) followed by goat anti-mouse IgG Alexa Fluor 488; rabbit anti-chromogranin A (stains enteroendocrine cells), rabbit anti-Ki-67 (stains replicating cells), and rabbit anti-mucin 2 (stains goblet cells) followed by goat anti-rabbit IgG Alexa Fluor 568; and goat anti-lysozyme (stains Paneth cells) followed by donkey anti-goat IgG Alexa Fluor 568; and Hoechst for nuclear staining. Scale bars = 20 μm.

(B) Transmission electron micrograph of mIECs grown on a transwell under ALI conditions for 6 days. The transwell membrane at the base of the cell monolayer is visible in the upper left (M). Epithelial cells displayed a typical columnar appearance with brush border (black arrows) and apical junctions (*) at the apical surface. Scale bar = 5 μm.

(C) Growth of *C. parvum* in ALI cultures. mIECs grown on transwells under ALI conditions were infected 3 days post top medium removal with 2 x 10^5 unfiltered *C. parvum* oocysts. Graph depicts qPCR measurement of *C. parvum* and mouse GAPDH equivalents (eq) from the same experiment. Mean ± S.D. of two transwells per time point. Replicate of experiment shown in Figure 1C.

(D) Immunofluorescence staining of oocyst wall forming bodies. ALI monolayers were infected with 1 μm-filtered *C. parvum* sporozoites 3 days post-medium removal and cultured for 7 days before processing for IFA. Monolayers were stained with mouse mAb OW50 followed by goat anti-mouse IgG Alexa 488; rabbit anti-*Toxoplasma* RH followed by goat anti-rabbit IgG Alexa 568; and Hoechst for nuclear staining. Scale bars = 5 μm.

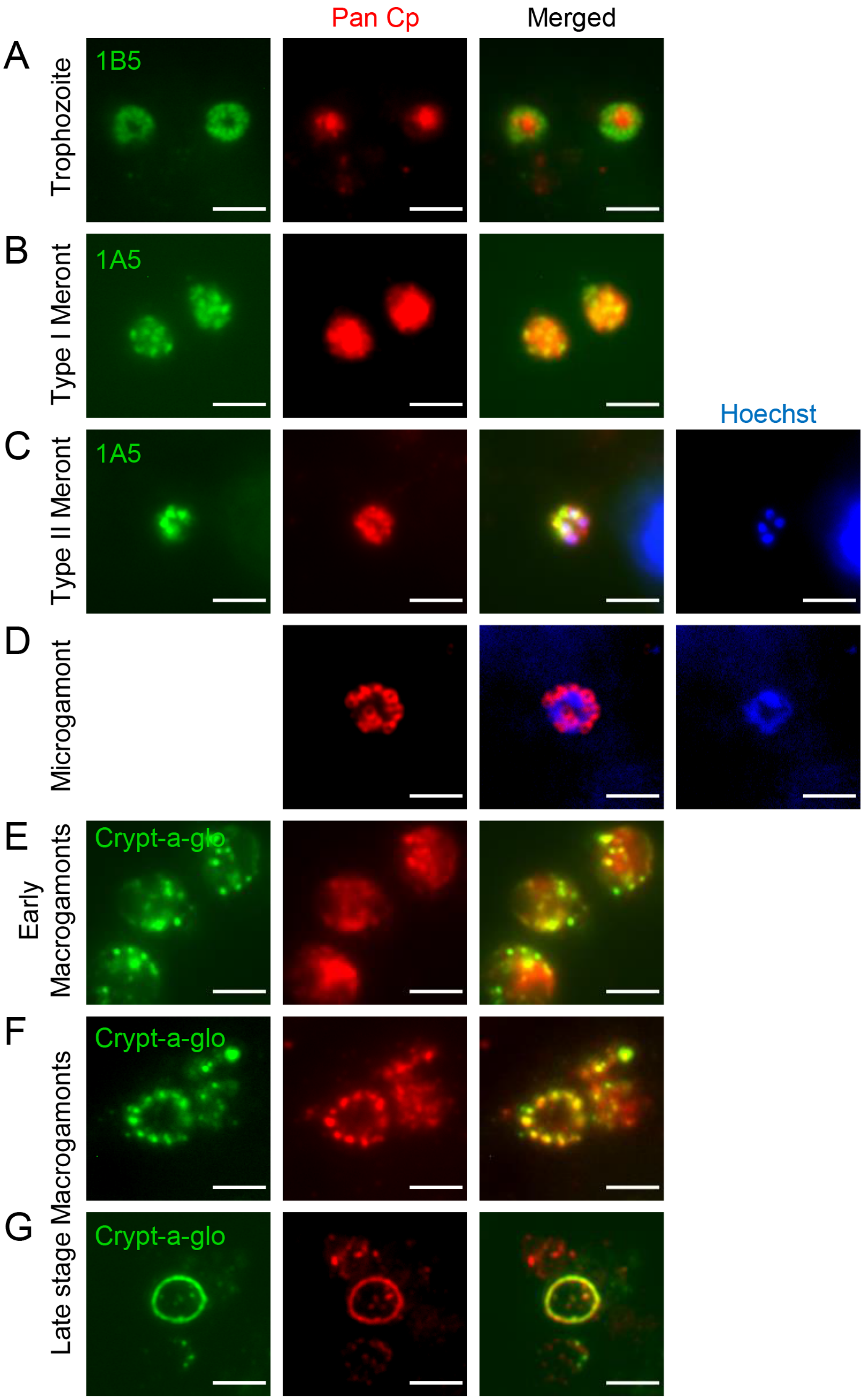

Pan Cp
Merged
Hoechst
A
Trophozoite
1B5
B
Type I Meront
1A5
C
Type II Meront
1A5
D
Microgamont
E
Early Macrogamonts
Crypt-a-glo
F
Macrogamonts
Crypt-a-glo
G
Late stage Macrogamonts
Crypt-a-glo

Figure S2 Individual channels for images of *C. parvum* asexual life cycle stages in ALI cultures. Related to Figure 1D.

(A-G) Infected ALI cultures were fixed and stained with Pan Cp followed by goat anti-rabbit IgG Alexa Fluor 568 to visualize all stages of *C. parvum* development.

(A) Trophozoites stained with mouse mAb 1B5, which recognizes the actin pedestal surrounding the parasite, followed by goat anti-mouse IgG Alexa Fluor 488.

(B) Type I meronts stained with mouse mAb 1A5, which recognizes the apical end of the eight mature merozoites, followed by goat anti-mouse IgG Alexa Fluor 488.

(C) Type II meront stained with mouse mAb 1A5, which recognizes the apical end of the four mature merozoites, followed by goat anti-mouse IgG Alexa Fluor 488. Hoechst staining confirms the presence of four nuclei.

(D) Microgamont stained with Hoechst to show the many small nuclei.

(E) Early macrogamonts display oocyst wall-forming bodies visualized with FITC-conjugated Crypt-a-glo^{TM}.

(F-G) In late stage macrogamonts, oocyst wall-forming bodies coalesce into a single ring as visualized with FITC-conjugated Crypt-a-glo^{TM}.

Scale bars = 3 μm

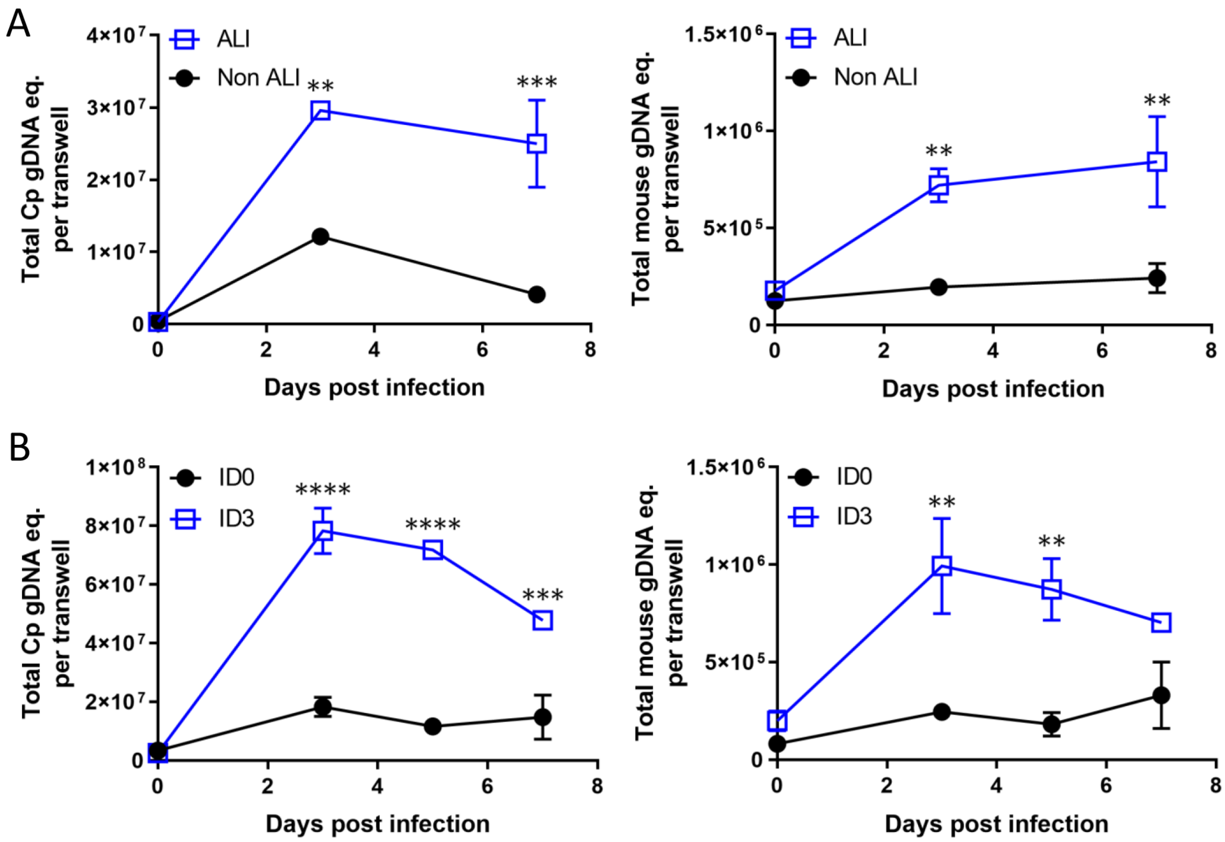

Figure S3 Effects of ALI growth conditions and time of infection post medium removal on *C. parvum* growth. Related to Figure 2.

(A-B) Comparison of *C. parvum* growth in (A) transwell cultures infected three days post medium removal (ALI) vs. transwells infected at the same time but with continuous top medium (Non ALI) or in (B) ALI cultures infected on day 0 (ID0) vs day 3 (ID3) post-medium removal. All transwells were infected with 2 x 10^5 unfiltered oocysts. Left graphs depict *C. parvum* genomic DNA equivalents (Cp gDNA eq.) and right graphs depict mouse genomic DNA equivalents (mouse gDNA eq.) from the same respective experiment as measured by qPCR of the respective GAPDH genes. Means ± S.D. from two transwells per time point. Replicate experiments of Figure 2A and 2B, respectively. Statistical analysis was conducted using two-way ANOVA corrected for multiple comparisons using the Sidak method, ** $P < 0.01$, *** $P < 0.001$, **** $P < 0.0001$.

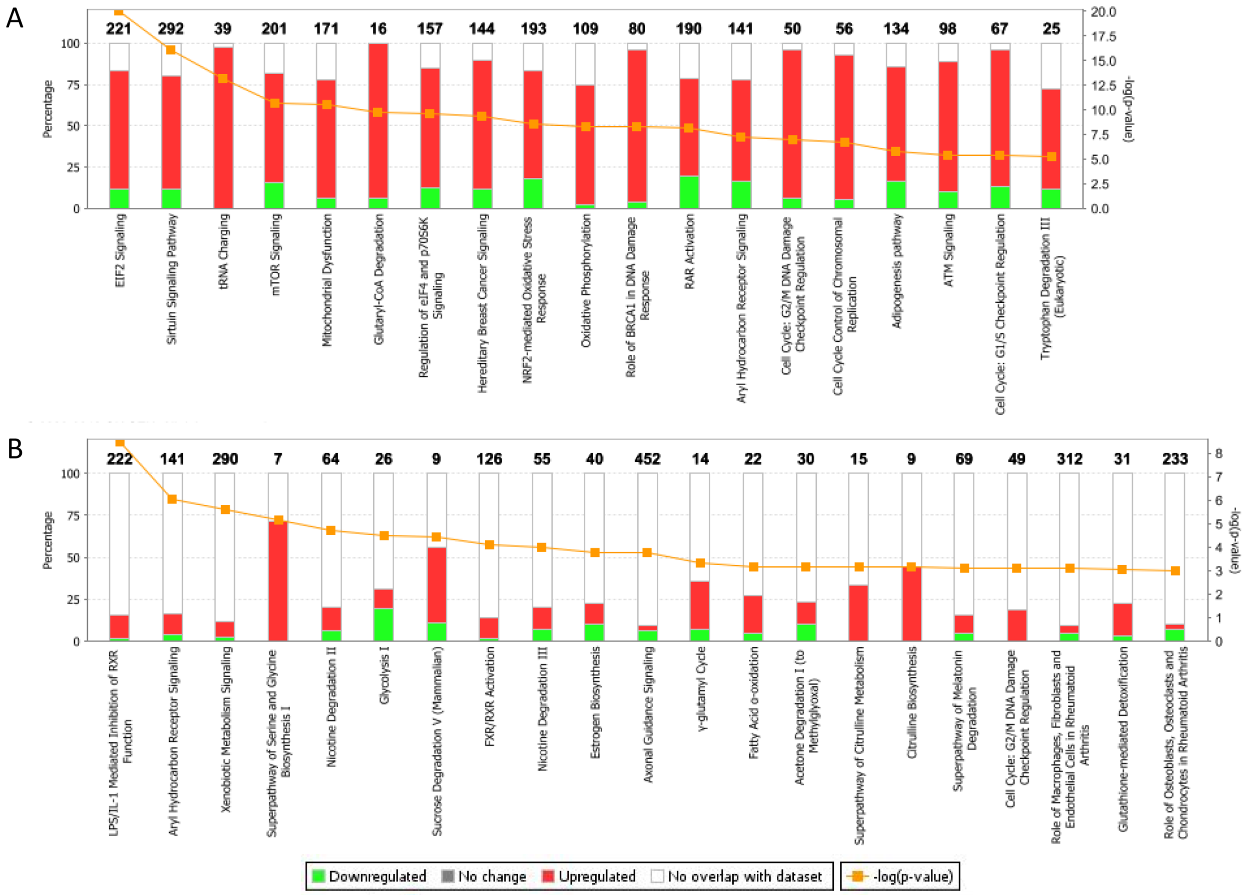

Figure S4 Analysis of major pathway differences in transcriptomes of ALI vs non-ALI cultures. Related to Figure 3.

(A-B) Bar chart of the percentage of genes upregulated (red) or downregulated (green) in biological pathways significantly enriched for differentially-expressed genes from day 3 ALI versus non-ALI samples when canonical pathway analysis is performed in Ingenuity Pathway Analysis (IPA) using (A) expression values for all genes as input or (B) only differentially expressed genes (false discovery rate < 0.05, absolute value fold change > 2) as input. In A, the major pathways identified by IPA analysis included increased expression of genes controlling protein translation (e.g. eIF2, tRNA, eIF4), signaling (e.g. mTOR, sirtuin signaling), cell cycle and replication (e.g. cell cycle, G2/M checkpoint, G1/S checkpoint, ATM signaling), and changes in metabolism (e.g. oxidative phosphorylation, adipogenesis, glutaryl coA degradation). In B, in addition to identifying similar changes in metabolism and cell proliferation noted above, induction of genes encoding nuclear receptor hormones (e.g. RAR, AHR, RXR, FxR, RxR) was seen in day 3 ALI cultures. Numbers in bold above each pathway indicates the

number of total genes in that pathway. Pathway significance cutoffs are $P < 0.00001$ for (A) and $P < 0.001$ for (B).

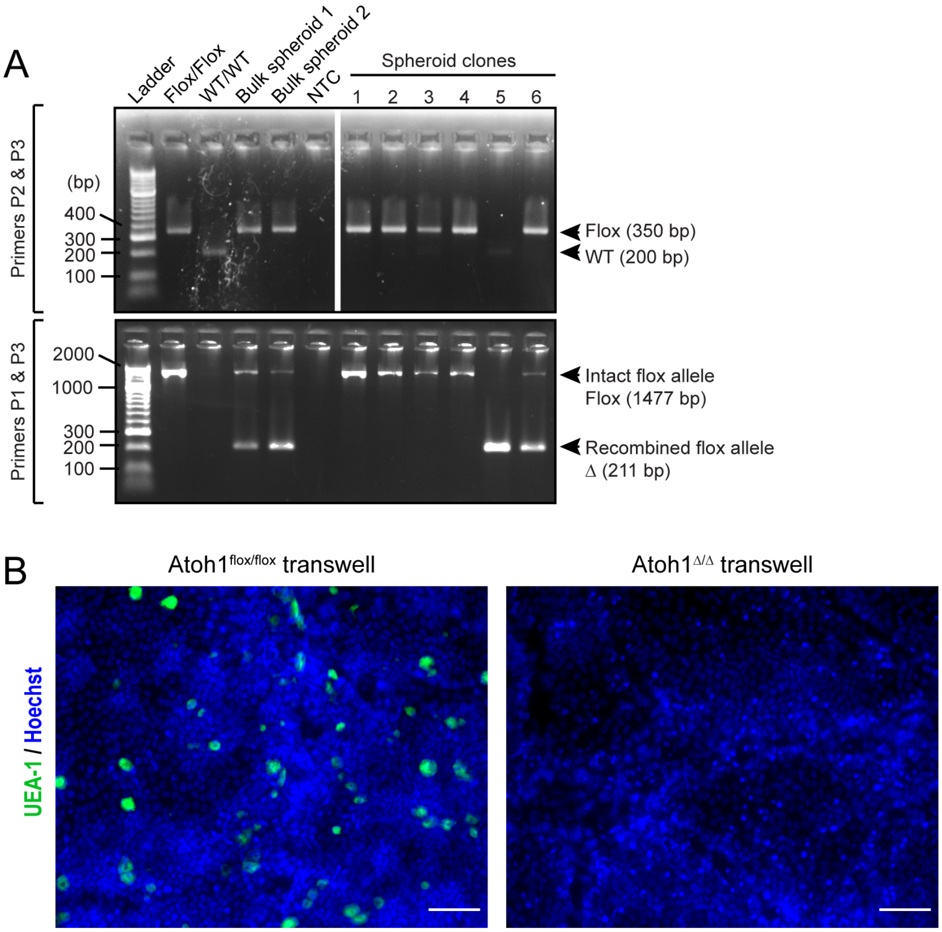

Figure S5 Validation of Atoh1-deficient spheroid line. Related to Figure 3.

(A) Representative images of ethidium bromide-stained agarose gels showing PCR genotyping products from the Atoh1-P2 and Atoh1-P3 (top) and Atoh1-P1 and Atoh1-P2 (bottom) primer combinations. Band sizes for the DNA ladder and expected PCR products are indicated in base pairs (bp). Positive control reactions used genomic DNA template from untreated Atoh1$^{flox/flox}$ and Atoh1$^{WT/WT}$ spheroids or bulk populations of Tat-Cre-treated spheroids (Bulk spheroid 1 & 2), which contained the Flox/Flox, Flox/Δ, and Δ/Δ genotypes. NTC, no template control. Six clonal jejunal spheroid lines are shown. Genotypes were determined to be as follows: Clones 1-4 = Flox/Flox; Clone 5 = Δ/Δ; Clone 6 = Flox/Δ.

(B) Whole-mount images of ALI transwells plated with clonal Atoh1$^{flox/flox}$ spheroids (left) or Atoh1$^{\Delta/\Delta}$ spheroids (right). Transwells were fixed and stained 7 days post media removal with an FITC-conjugated UEA-1 lectin (detects mucus-secreting goblet cells) and Hoechst DNA stain. Scale bar = 50 μm

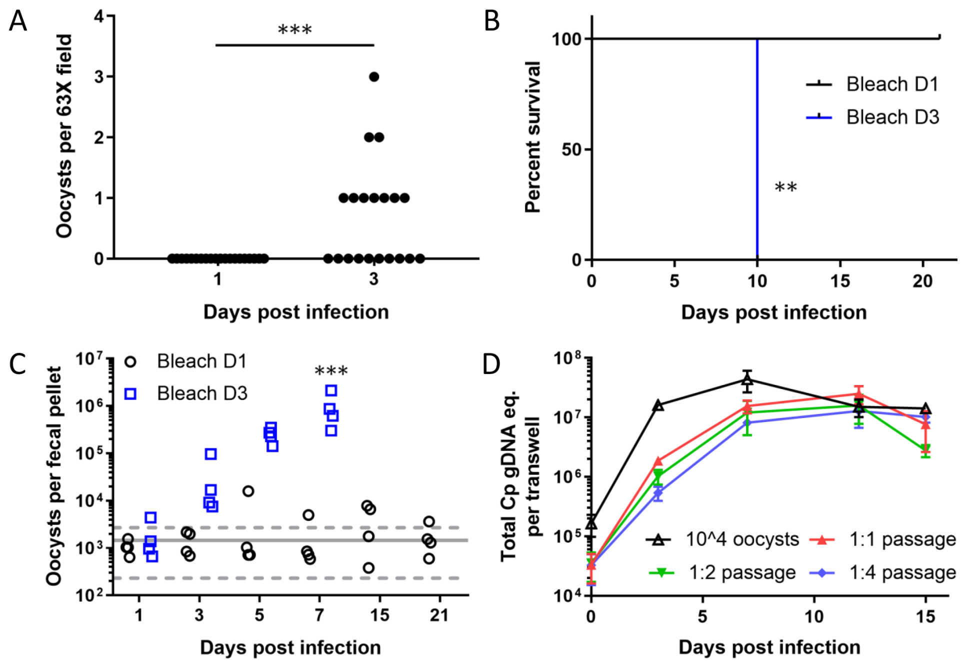

Figure S6 Infectivity of oocysts produced in ALI cultures. Related to Figure 5

(A-D) ALI transwell cultures were infected with 1 μm-filtered sporozoites. On the indicated days post infection, transwells were bleached, washed and (A) adhered to PLL-coated coverslips, (B-C) gavaged into Ifngr1$^{-/-}$ mice or (D) used to infect fresh ALI transwells.

(A) Detection of oocysts in ALI cultures treated with bleach. Coverslips with day 1 or day 3 bleached material were stained with Pan Cp followed by goat anti-rabbit Alexa Fluor 568 and Crypt-a-glo^{TM} directly conjugated to FITC. Each data point is the number of oocysts in a separate field from a single experiment. Replicate experiment of Figure 5A. Data was analyzed using a Mann-Whitney U test. *** $P < 0.001$.

(B) Survival curves of the mice infected with day 1 versus day 3 bleached material. Data was analyzed using the log-rank (Mantel-Cox) test. ** $P < 0.01$.

(C) Number of *C. parvum* oocysts per fecal pellet (one pellet per mouse) as measured by qPCR. The gray line represents the mean value of both groups for day 1, the dotted lines denote the standard deviations. Data was analyzed using a two-way ANOVA comparing the mean of each group across all time points, corrected for multiple comparisons using the Sidak method *** $P < 0.001$.

(D) Passage of bleached ALI monolayers. Naïve ALI monolayers were infected with day 3 bleached material at a 1:1, 1:2 or 1:4 old to new transwell passage ratio or with 10^4 calf-derived oocysts. Total *C. parvum* genome eq. per transwell was measured by qPCR. Data plotted as mean ± S.D. from two transwells per timepoint. Replicate experiment of Figure 5F.

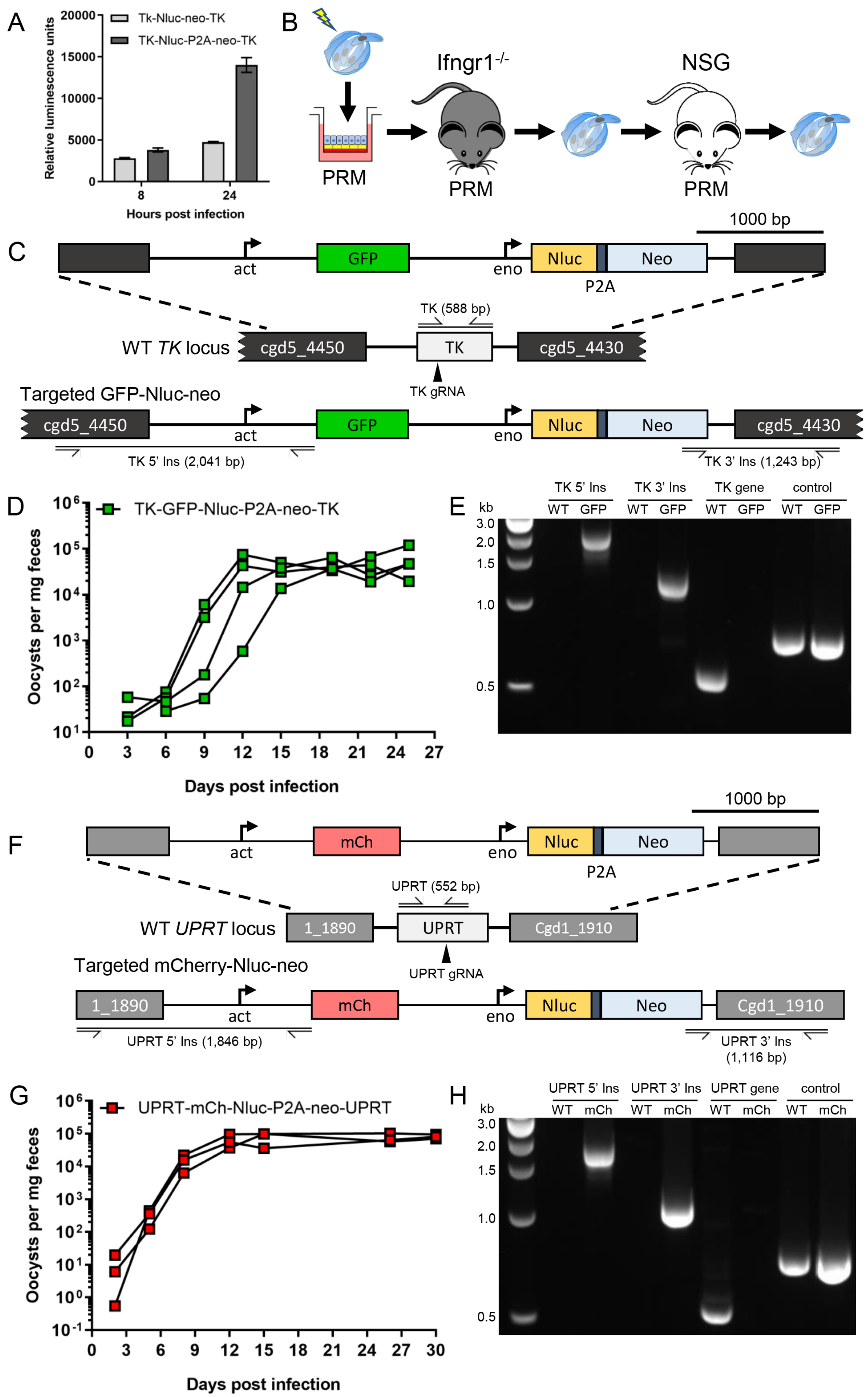
A
Relative luminescence units
20000
15000
10000
5000
0
Tk-Nluc-neo-TK
TK-Nluc-P2A-neo-TK
8
24
Hours post infection
B
PRM
Ifngr1-/-
PRM
NSG
PRM
C
1000 bp
act
GFP
eno
Nluc
Neo
P2A
TK (588 bp)
WT TK locus
cgd5_4450
TK
cgd5_4430
TK gRNA
Targeted GFP-Nluc-neo
cgd5_4450
act
GFP
eno
Nluc
Neo
cgd5_4430
TK 5' Ins (2,041 bp)
TK 3' Ins (1,243 bp)
D
TK-GFP-Nluc-P2A-neo-TK
Oocysts per mg feces
Days post infection
E
kb
TK 5' Ins
TK 3' Ins
TK gene
control
WT GFP WT GFP WT GFP WT GFP
3.0
2.0
1.5
1.0
0.5
F
1000 bp
act
mCh
eno
Nluc
Neo
P2A
UPRT (552 bp)
WT UPRT locus
1_1890
UPRT
Cgd1_1910
UPRT gRNA
Targeted mCherry-Nluc-neo
1_1890
act
mCh
eno
Nluc
Neo
Cgd1_1910
UPRT 5' Ins (1,846 bp)
UPRT 3' Ins (1,116 bp)
G
UPRT-mCh-Nluc-P2A-neo-UPRT
Oocysts per mg feces
Days post infection
H
kb
UPRT 5' Ins
UPRT 3' Ins
UPRT gene
control
WT mCh WT mCh WT mCh WT mCh
3.0
2.0
1.5
1.0
0.5

Figure S7 Amplification of transgenic parasites in immunocompromised mice. Related to Figure 6.

(A) Relative luminescence of *C. parvum* parasites 8 or 24 hrs after transfection with an Nluc-neo plasmid either with (dark gray) or without (light gray) the P2A skip peptide inserted between the Nluc and neo coding sequences.

(B) Following selection for transgenic parasites in ALI cultures, oocysts were amplified by passage in immunodeficient mice. Infected transwells were syringed lysed at day 4-6 post-infection and gavaged into Ifngr1$^{-/-}$ mice treated with paromomycin in the drinking water (16 g/L). A second round of amplification was conducted in NOD *Scid* gamma (NSG) immunodeficient mice. Oocysts were purified from NSG fecal pellets.

(C) Diagram of the TK-GFP-Nluc-P2A-neo-TK targeting plasmid and its insertion into the *tk* locus (cgd5_4440) when transfected with a CRISPR/Cas9 plasmid containing a TK gRNA.

(D) The number of oocysts per mg of feces was measured by qPCR. Each data point represents a single pellet and each connecting line represents an individual NSG mouse infected with TK-GFP-Nluc-P2A-neo-TK parasites.

(E) PCR confirmation that TK-GFP-Nluc-P2A-neo-TK oocysts amplified in mice have the correct insert and lack the *tk* gene.

(F) Diagram of the UPRT-mCh-Nluc-P2A-neo-UPRT targeting plasmid and its insertion into the *uprt* locus (cgd1_1900) when transfected with a CRISPR/Cas9 plasmid containing a UPRT gRNA.

(G) The number of oocysts per mg of feces was measured by qPCR. Each data point represents a single pellet and each connecting line represents an individual NSG mouse infected with UPRT-mCh-Nluc-P2A-neo-UPRT parasites.

(H) PCR confirmation that UPRT-mCh-Nluc-P2A-neo-UPRT oocysts amplified in mice have the correct insert and lack the *uprt* gene.

Table S1 Meiosis genes in *C. parvum.* Related to Figure 6

Meiosis Genes	*C. parvum* Gene ID	CryptoDB gene annotation	Annotated homolog as query for blastp	blastp query coverage	blastp E-value	blastp % identity
Spo11	cgd8_1350	Spo11/ DNA topoisomerase 6 subunit A	*P. falciparum* PF3D7_1217100	86%	1E-39	32%
Mnd1	cgd1_840	Meiotic nuclear division protein 1	*P. falciparum* PF3D7_1461500	30%	3E-08	45%
Dmc1	cgd7_1690	Rad51/DMC1/RadA DNA repair protein	*P. falciparum* PF3D7_0816800	100%	4E-173	65%
Rad51	cgd5_410	Rad51	*T. gondii* TGME49_272900	91%	0	73%
Rec8/rad21	cgd8_1020	Rad21/Rec8-like				
Rec8/rad21	cgd7_290	Rad21/Rec8-like				
Hop1	cgd5_1750	HORMA domain containing protein	*S. cerevisiae* Hop1p	39%	6E-22	30%
Hop2	cgd2_510	Homologous-pairing protein 2	*S. cerevisiae* Hop2p	28%	3E-06	35%
Msh2	cgd8_3950	MutS-like ABC Atpase	*P. falciparum* PF3D7_0706700	89%	4E-140	33%
Msh6	cgd8_370	DNA mismatch repair protein MutS family domain containing protein	*P. falciparum* PF3D7_0505500	73%	0	38%
Msh4	absent	N/A				
Msh5	absent	N/A				
